# SREBP1c‐Mediated Transcriptional Repression of YME1L1 Contributes to Acute Kidney Injury by Inducing Mitochondrial Dysfunction in Tubular Epithelial Cells

**DOI:** 10.1002/advs.202412233

**Published:** 2024-12-16

**Authors:** Wang Xin, Jie Zhou, Yuzhu Peng, Shuiqin Gong, Wenhao Liao, Yaqin Wang, Xixin Huang, Yang Mao, Mengying Yao, Shaozong Qin, Jiachuan Xiong, Yan Li, Qigang Lan, Yinghui Huang, Jinghong Zhao

**Affiliations:** ^1^ Department of Nephrology Chongqing Key Laboratory of Prevention and Treatment of Kidney Disease Chongqing Clinical Research Center of Kidney and Urology Diseases Xinqiao Hospital Army Medical University (Third Military Medical University) Chongqing 400037 China; ^2^ Department of Oncology Southwest Cancer Center Southwest Hospital Army Medical University Chongqing 400038 China; ^3^ Clinical Medical Research Center Xinqiao Hospital Army Medical University Chongqing 400037 China

**Keywords:** AKI, Mitochondrial dysfunction, SREBP1c, YME1L1

## Abstract

Acute kidney injury (AKI) is a prevalent clinical syndrome with high morbidity and mortality. Accumulating studies suggest mitochondrial dysfunction as the typical characteristics and key process of AKI, but the underlying mechanism remains elusive. The YME1‐like 1 (YME1L1) ATPase, an inner mitochondrial membrane protein, is screened and identified to be downregulated in renal tubular epithelial cells of various mouse models and patients of AKI. Dramatically, restoration of YME1L1 expression significantly alleviates cisplatin‐induced AKI and subsequent chronic kidney disease (CKD) through attenuating mitochondrial dysfunction via maintaining optic atrophy 1 (OPA1)‐mediated mitochondrial energy metabolism homeostasis. Mechanistically, the upregulated expression of sterol regulatory element binding transcription factor 1c (SREBP1c) is demonstrated to be responsible for cisplatin‐mediated transcriptional inhibition of YME1L1 via directly binding to its promoter region. Moreover, cisplatin‐induced methyltransferase‐like 3 (METTL3)‐mediated m6A modification enhances SREBP1c mRNA stability, thereby upregulating its expression. Notably, both depletion of SREBP1c and renal tubule‐specific overexpression of YME1L1 markedly ameliorate cisplatin‐induced AKI and its transition to CKD. Taken together, these findings suggest that METTL3‐mediated SREBP1c upregulation contributes to AKI and its progression to CKD through disrupting mitochondrial energy metabolism via transcriptionally suppressing YME1L1. Targeting the SREBP1c/YME1L1 signaling may serve as a novel therapeutic strategy against AKI.

## Graphical abstract

This study reveals that METTL3‐mediated SREBP1c upregulation contributes to AKI and its progression to CKD through inducing mitochondrial energy metabolism dysfunction via transcriptionally suppressing YME1L1. Both overexpression of YME1L1 and knockout of SREBP1c ameliorate mitochondrial energy metabolism dysfunction in RTECs, thereby retarding AKI and its progression to CKD. Targeting the SREBP1c/YME1L1 signaling may be a novel therapeutic strategy against AKI.

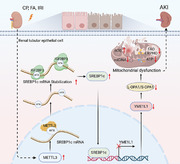

## 1 Introduction

Acute kidney injury (AKI) is a life‐threatening clinical syndrome with high morbidity and mortality, occurring in 10–15% of hospitalizations and over 50% of patients in intensive care unit.^[^ ^1^ ^]^ AKI can be triggered by a variety of causes, including nephrotoxic drugs, sepsis, and ischemia reperfusion injury (IRI) due to severe infections.^[^ ^1c^ ^]^ Cisplatin is a first‐line drug frequently used in solid tumors, which generally causes significant kidney injury. It has been extensively reported to damage the proximal tubular epithelial cells (PTECs), thereby causing AKI.^[^ ^2^ ^]^ In addition, increasing evidence suggests that maladaptive and incomplete renal repair after AKI results in tubular atrophy and interstitial fibrosis, ultimately contributing to the development of chronic kidney disease (CKD).^[^ ^3^ ^]^ However, the underlying mechanisms of AKI and its progresses to CKD remain elusive.

The kidney, a highly metabolically active organ, depends on numerous mitochondria for energy production essential for blood waste clearance and fluid and electrolyte balance.^[^ ^4^ ^]^ Thus, mitochondrial damage can directly lead to kidney injury, and accumulating studies suggest mitochondrial dysfunction as one of the most typical characteristics and key processes of AKI, even though the interventions are limited. Recent studies demonstrated that during AKI, mitochondrial homeostasis, and dynamics are imbalanced, which includes depletion of adenosine triphosphate (ATP), excessive reactive oxygen species (ROS) production, and destruction of oxidative phosphorylation (OXPHO) and fatty acid oxidation (FAO) that further lead to renal tubular apoptosis and subsequent interstitial fibrosis.^[^ ^5^ ^]^ Therefore, targeting mitochondrial dysfunction may serve as a potential therapeutic target for AKI.

YME1‐like 1 (YME1L1), a member of the AAA ATPase family, is an ATP‐dependent metalloprotease encoded by the nuclear genome embedded in the inner mitochondrial membrane, which can mediate the remodeling, unfolding and degradation of mitochondrial proteins, making it capable of stabilizing mitochondrial structure and maintaining mitochondrial function.^[^ ^6^ ^]^ Previous studies have shown that YME1L1 knockdown activates the ubiquitin‐proteasome system, mediated by the forkhead box O3A gene (FoxO3a) in muscle, leading to mitochondrial dysfunction and muscle atrophy via down‐regulation of genes related to mitochondrial biosynthesis.^[^ ^7^ ^]^ Additionally, YME1L1 participates in regulating various pathophysiological processes, including immune regulation, neurological dysfunction, and tumorigenesis.^[^ ^8^ ^]^ However, the potential roles and exact mechanisms of YME1L1 in the kidney remain unclear.

In this study, we screened and demonstrated that YME1L1 was downregulated in PTECs under AKI conditions. Renal tubule specific restoration of YME1L1 expression attenuated cisplatin‐induced AKI and subsequent CKD via restoring mitochondrial energy metabolism homeostasis. Further, SREBP1c was identified as a novel regulator of YME1L1 and was responsible for cisplatin‐mediated transcriptional suppression of YME1L1 via binding to the promoter region of YME1L1. It was demonstrated that cisplatin‐induced METTL3‐mediated m6A modification increased the mRNA stability of SREBP1c, thereby upregulating its expression, while depletion of SREBP1c markedly ameliorated cisplatin‐induced AKI and its progression to CKD. Therefore, targeting the SREBP1c/YME1L1 signaling may serve as a novel therapeutic strategy for AKI treatment.

## 2 Results

### 2.1 YME1L1 Expression is Downregulated in PTECs under AKI Conditions

**Figure 1:**
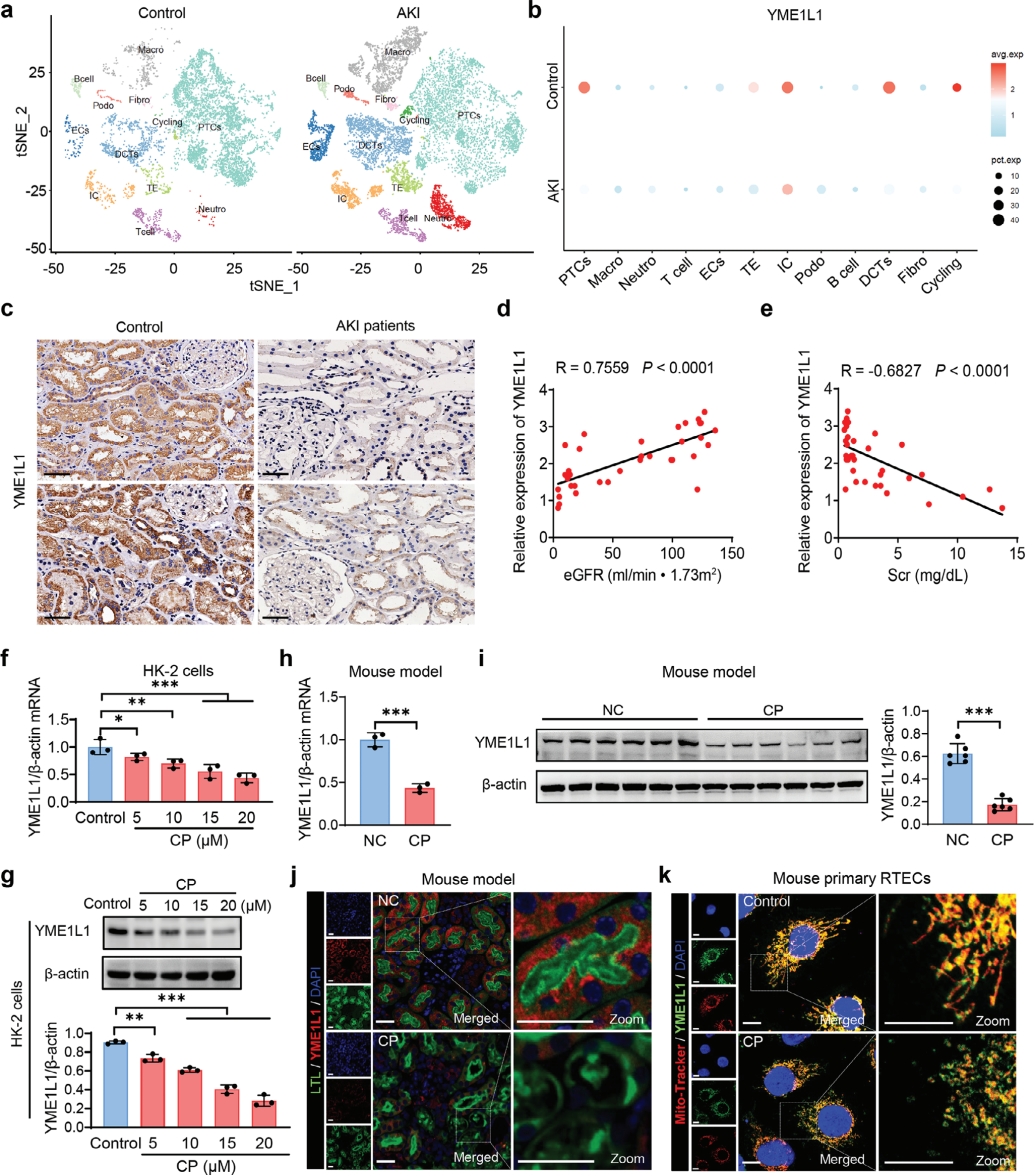
YME1L1 is downregulated in AKI. a) Different cell types and clusters were identified in the kidney tissues of AKI mice through snRNA‐seq. The t‐distributed stochastic neighbor embedding (t‐SNE) plot illustrates the major cell clusters. ECs: endothelial cells; Macro: macrophage; Fibro: Fibroblast; Neutro: neutrophil; PTCs: proximal tubular cells; DCTs: distal convoluted tubule; IC: intercalated cell; TE: transitional epithelia; Cycling: Proliferating cells. b) Dot plot of YME1L1 expression in each cell population. c) Representative images of IHC staining of YME1L1 in the renal biopsies from AKI patients (*n* = 21) and para‐carcinoma tissues of renal nephrectomy samples from patients with kidney cancer (*n* = 15). Scale bar, 50 µm. d,e) Correlation analysis between YME1L1 staining intensity and renal function. f,g) The levels of YME1L1 were analyzed using qPCR and Western blot analyses in HK‐2 cells incubated with different concentrations of Cisplatin (CP) for 24 h (*n* = 3). h,i) The expression of YME1L1 was analyzed by qPCR and Western blot analysis in an AKI mouse model induced by CP (25 mg kg^−1^). (*n* = 8 mice in each group). j) Representative immunofluorescence staining of YME1L1 (red) expression in kidney tissues from mice treated with negative control (NC) or CP for 3 days. Lotus lectin (LTL, green) was used to label proximal tubules. Scale bar, 20 µm. k) Representative immunofluorescence staining of YME1L1 (green) expression in murine primary isolated RTECs with control or 20 µm CP treatment for 24 h. Mito‐Tracker (red) was used to label mitochondria. Scale bar, 10 µm. Data are shown as means ± SD and were analyzed by Spearman's rank correlation test (d and e), one‐way ANOVA (f and g) or two‐tailed unpaired Student's *t*‐test (h and i). *P < 0.05, **P < 0.01, and ***P < 0.001.

To identify key contributors to AKI, we performed bioinformatics analysis using RNA sequencing (RNA‐seq) and single nucleus RNA sequencing (snRNA‐Seq) data from kidneys of sham and AKI mice (GSE87025 and GSE197266), which identified YME1L1 as one of the most downregulated genes (**Figure** **1**a,b; Figure S1a, Supporting Information). Then, qPCR and Western blot demonstrated that YME1L1 was highly expressed in normal mouse kidney (Figure S1b,c, Supporting Information). To validate the RNA‐seq findings, renal biopsy specimens from 21 patients with AKI and 15 controls with no evident kidney injury were enrolled in this study (Table S1, Supporting Information). Immunohistochemical (IHC) staining revealed that YME1L1 expression was suppressed in renal tissues of AKI patients and positively correlated with renal function (Figure 1c–e). YME1L1 expression was also suppressed in human renal proximal tubular epithelial cells (HK‐2) following cisplatin and hypoxia‐reoxygenation (HR) treatment (Figure 1f,g; Figure S1d–f, Supporting Information). To further explore the expression and potential roles of YME1L1 in AKI, we constructed three AKI mouse models,^[^ ^9^ ^]^ and found that YME1L1 expression was markedly inhibited at both transcriptional and translational levels in the kidney tissues of AKI mice induced by cisplatin, IRI, and folic acid (FA), compared with control mice (Figure 1h,i; Figure S2a–o, Supporting Information). In addition, we also evaluated the cellular localization of YME1L1 in the kidney. It was demonstrated that YME1L1 was localized in the mitochondria of murine primary isolated renal tubular epithelial cells (RTECs) and was abundantly expressed in the proximal tubules (Figure 1j,k; Figure S2p, Supporting Information), further indicating its potential involvement in AKI. Collectively, these findings suggest that YME1L1 expression is downregulated during AKI.

### 2.2 Restoration of YME1L1 Attenuates Cisplatin‐Induced Mitochondrial Dysfunction In Vitro

**Figure 2:**
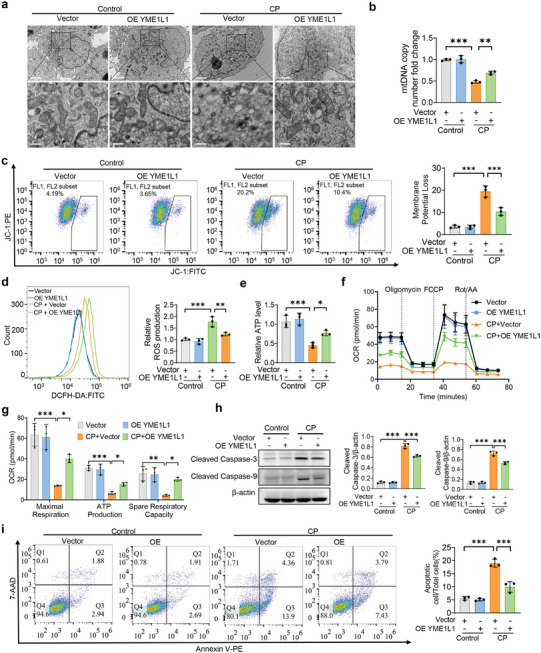
YME1L1 attenuates mitochondrial dysfunction induced by cisplatin in vitro. a) YME1L1 overexpression plasmids or vector were transfected into HK‐2 cells and then they were incubated with control or 20 µm CP for 24 h. These cells were collected for TEM observation (**a**, scale bar, 2 µm (top) and 0.5 µm (bottom)). b–i) Cells in (a) were collected to analyze the relative mtDNA copy number (b) (*n* = 3), monomer and aggregate JC‐1 (c) (*n* = 3), production of ROS (d) (*n* = 3), relative ATP levels (e) (*n* = 3), oxygen consumption rate (OCR) (f and g) (*n* = 3), the expressions of cleaved caspase‐3 and cleaved caspase‐9 (h) (*n* = 3), and Annexin V‐PE/7‐AAD staining by flow cytometry analysis and quantification of apoptotic cells (**i**) (*n* = 3). Data are shown as means ± SD and were analyzed by one‐way ANOVA (b‐i). *P < 0.05, **P < 0.01, and ***P < 0.001.

Given the crucial role of YME1L1 in stabilizing mitochondrial structure,^[^ ^6^ ^]^ and mitochondrial injury has been identified as a typical characteristic of AKI, we first explored the role of YME1L1 in a cisplatin‐induced AKI model in vitro. Transmission electron microscopy (TEM) observations showed that cisplatin‐induced mitochondrial structural damages with fragmentation, swelling, and disruption of cristae were significantly improved by overexpression of YME1L1 in HK‐2 cells transfected with YME1L1 overexpression plasmids (**Figure** **2**a; Figure S3a,b, Supporting Information). Further, overexpression of YME1L1 significantly reversed cisplatin‐induced reduction of mitochondrial DNA (mtDNA), depolarization of membrane potential, elevation of ROS production, and ATP levels (Figure 2b–e). Then, we measured the oxygen consumption rate (OCR) with seahorse, and it was found that YME1L1 overexpression attenuated the decreased OCR, maximal respiratory capacity, ATP production, and spare respiratory capacity in cisplatin‐incubated HK‐2 cells (Figure 2f,g). Additionally, Western blot and flow cytometry demonstrated that overexpression of YME1L1 rescued cisplatin and HR‐induced apoptosis, and reduced the upregulation of kidney injury markers (kidney injury molecule 1 (KIM1) and neutrophil gelatinase‐associated lipocalin (NGAL)) in HK‐2 cells (Figure 2h,i; Figure S3c–f, Supporting Information). These results indicate that restoration of YME1L1 expression significantly attenuates cisplatin‐induced mitochondrial dysfunction in vitro.

### 2.3 YME1L1 Ameliorates Cisplatin‐Induced Mitochondrial Dysfunction through Restoring OPA1‐Mediated Mitochondrial Energy Metabolism Homeostasis

**Figure 3:**
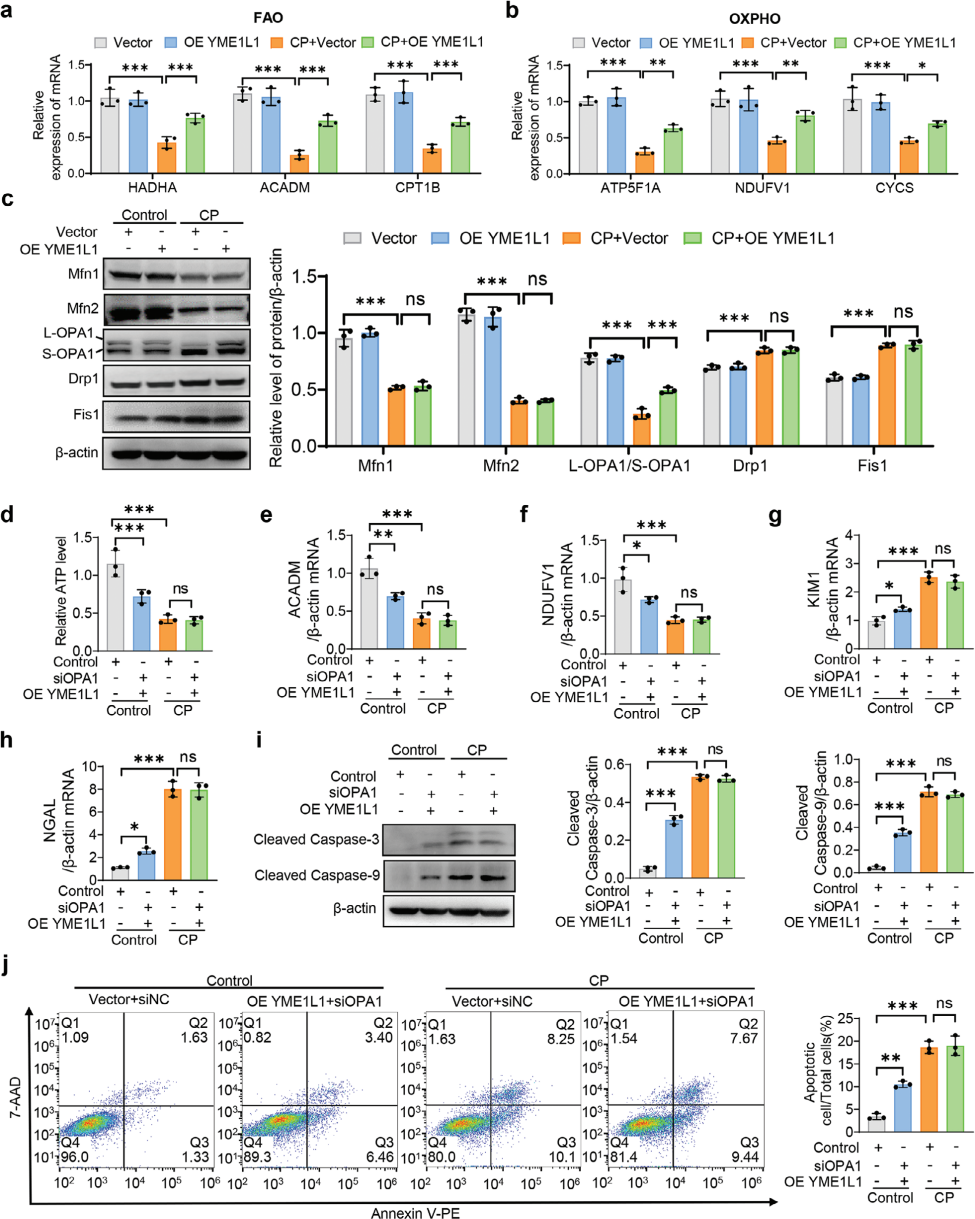
YME1L1 ameliorates cisplatin‐induced mitochondrial dysfunction via restoring OPA1‐mediated mitochondrial energy metabolism homeostasis. a,b) qPCR analysis of FAO‐related genes (hydroxyacyl‐CoA dehydrogenase trifunctional, HADHA; acyl‐CoA dehydrogenase medium chain, ACADM; carnitine palmitoyltransferase 1B; CPT1B) and OXPHO‐related genes (ATP synthase F1 subunit alpha, ATP5F1A; NADH: ubiquinone oxidoreductase core subunit V1, NDUFV1; cytochrome c, somatic, CYCS) expression (a and b) in HK‐2 cells, transfected with vector or YME1L1 overexpression plasmids in combination with control or 20 µm CP treatment for 24 h (*n* = 3). c) The expression levels of Mfn1, Mfn2, L‐OPA1/S‐OPA1, Drp1, and Fis1 in cells in (a) were analyzed by Western blot. d–j) HK‐2 cells were transfected with vector or OE YME1L1 and siNC or siOPA1, and then exposed to control or 20 µm CP for 24 h for determination of relative ATP levels (d) (*n* = 3), the mRNA expressions of ACADM, NDUFV1, KIM1, and NGAL e–h) (*n* = 3), Western blot analysis of cleaved caspase‐3 and cleaved caspase‐9 expression i) (*n* = 3) and flow cytometry analysis of apoptotic cells j) (*n* = 3). Data are shown as means ± SD and were analyzed by one‐way ANOVA (a–j). ns: no significance. *P < 0.05, **P < 0.01, and ***P < 0.001.

To investigate the mechanism how mitochondrial ATP levels were reduced, we measured the FAO and OXPHO‐related gene expression and demonstrated that overexpression of YME1L1 significantly rescued the cisplatin‐induced decrease in FAO and OXPHO‐related genes (**Figure** **3**a,b). Noteworthy, we screened the mitochondrial fusion and fission‐related genes, and found that overexpression of YME1L1 only rescued cisplatin‐mediated downregulation of optical atrophy 1 (OPA1), without affecting mitofusin 1 (Mfn1), mitofusin 2 (Mfn2), mitochondrial fission 1 (Fis1) or dynamin related protein 1 (Drp1) (Figure 3c). Further studies demonstrated that YME1L1 improved cisplatin‐induced mitochondrial dysfunction via balancing long isoforms of OPA1 (L‐OPA1)/ short isoforms of OPA1 (S‐OPA1) expression in HK‐2 cells, since siRNAs‐mediated knockdown of OPA1 reversed the protective effect of YME1L1, as evidenced by decreased ATP levels, suppressed expressions of FAO and OXPHO‐related genes, elevated expressions of KIM1 and NGAL, and increased apoptosis (Figure 3d–j; Figure S4a, Supporting Information). Additionally, YME1L1 knockdown significantly downregulated L‐OPA1/S‐OPA1 expression (Figure S4b, Supporting Information). These results reveal that YME1L1 alleviates cisplatin‐induced mitochondrial dysfunction through restoring OPA1‐mediated mitochondrial energy metabolism homeostasis.

### 2.4 SREBP1c Represses the Transcription of YME1L1 via Directly Binding to its Promoter Region

**Figure 4:**
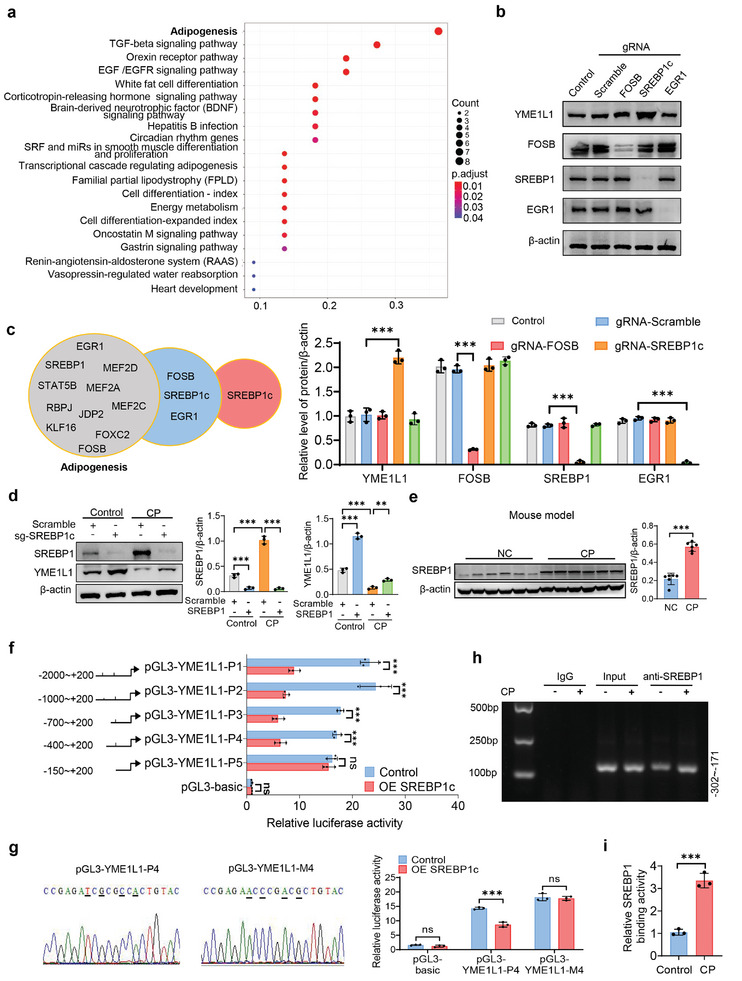
SREBP1c suppresses YME1L1 transcription via directly binding to its promoter region. a) GO analysis of the biological processes involved in the predicted transcription factors by cluster profiler. b) HK‐2 cells were transfected with sgRNA (Scramble, FOSB, SREBP1c, or EGR1), and collected for Western blot analysis of YME1L1, FOSB, SREBP1, and EGR1 (*n* = 3). c) Schematic diagram of transcription factor screening. d) HK‐2 cells were transfected with sgRNA (Scramble or SREBP1c) in combination with control or 20 µm CP treatment for 24 h, and then YME1L1 and SREBP1 expression was analyzed by Western blot (*n* = 3). e) SREBP1 expression was analyzed by Western blot in control or AKI mouse model induced by CP (25 mg kg^−1^) (*n* = 6). f) HK‐2 cells were co‐transfected with pRL‐TK vector and pGL3‐basic or recombinant plasmids containing various YME1L1 promoter regions together with control or OE SREBP1c. Then Cells were collected for the dual luciferase reporter gene assay (*n* = 3). g) HK‐2 cells were co‐transfected with pRL‐TK vector and pGL3‐YME1L1‐P4 or pGL3‐YME1L1‐M4 (containing mutant bases of pGL3‐YME1L1‐P4 underlined in the sequencing data), treated with control or OE SREBP1c for luciferase assays (*n* = 3). h,i) ChIP assay in HK‐2 cells with treatment of control or CP for 24 h. IgG served as the negative control. The primers covering the YME1L1 promoter region (−302–−171) were used to amplify the precipitated DNA by PCR (h) and qPCR (i) (*n* = 3). Data are shown as means ± SD and were analyzed by two‐tailed unpaired Student's *t*‐test (e–g and i) or one‐way ANOVA (b and d). ns: no significance. **P < 0.01, ***P < 0.001.

To elucidate the mechanism of cisplatin‐mediated downregulation of YME1L1, we first demonstrated that cisplatin had no significant effects on the mRNA stability and protein degradation of YME1L1 (Figure S5, Supporting Information). Therefore, bioinformatics was applied to predict potential transcription factors in the promoter region of the *YME1L1* gene (Table S2, Supporting Information). Subsequent GO enrichment and qPCR screening showed that adipogenesis‐related genes, including Fosb proto‐oncogene (FOSB), early growth response 1 (EGR1), and SREBP1c, were significantly up‐regulated by cisplatin both in vitro and in vivo (**Figure** **4**a; Figure S6a–c, Supporting Information). Of note, CRISPR/Cas9 genome editing‐mediated single‐guide RNAs (sgRNAs) targeting SREBP1c, rather than FOSB or EGR1, upregulated the YME1L1 expression (Figure 4b–e; Figure S6d, Supporting Information). Interestingly, SREBP1c expression was significantly elevated, while YME1L1 was downregulated in a time‐dependent manner in HK‐2 cells and mouse kidney tissues treated with cisplatin (Figures S1f, S2n, and S6e,f, Supporting Information). As expected, the sgRNA targeting SREBP1c also upregulated YME1L1 expression following 6 h of cisplatin treatment (Figure S6g, Supporting Information). However, the classical lipogenic SREBP1c target genes (fatty acid synthase (FASN) and acetyl CoA carboxylase 1 (ACC1))^[^ ^10^ ^]^ were only mildly elevated or unchanged during the pre‐AKI phase until day 4 (Figure S6h,i, Supporting Information). These results indicate that SREBP1c has a strong inhibitory effect on YME1L1 expression in the early stage of AKI.

Based on the predicted SREBP1c binding site in the YME1L1 promoter domain (Table S3, Supporting Information), we speculated that SREBP1c could repress YME1L1 transcription by directly binding to its promoter region. The recombinant luciferase reporter assay revealed that SREBP1c overexpression (OE SREBP1c) markedly inhibited the luciferase activity of pGL3‐YME1L1‐P1–P4, while having no effect on pGL3‐YME1L1‐P5 (Figure 4f), implying that the SREBP1c‐response element is present in the sequence of −400 to −150 relative to the transcription start site. Bioinformatic analysis predicted that the binding sites might include the sequence spanning −214 to −205 (ATCGCGCCAC) (Table S3, Supporting Information). As anticipated, mutation of pGL3‐YME1L1‐P4 (−214 to −205) abolished OE SREBP1c‐mediated inhibitory activity on the YME1L1 promoter region (Figure 4g). Further, ChIP assays were conducted, which showed a direct binding of SREBP1c to YME1L1 promoter region (−302 to −171, rather than −1358 to −1161), which was significantly enhanced by cisplatin administration (Figure 4h,i; Figure S6j, Supporting Information). These findings suggest that cisplatin‐induced upregulation of SREBP1c inhibits YME1L1 transcription by directly binding to its promoter region.

### 2.5 Knockout of SREBP1c Alleviates Cisplatin‐Induced Mitochondrial Dysfunction In Vitro

**Figure 5:**
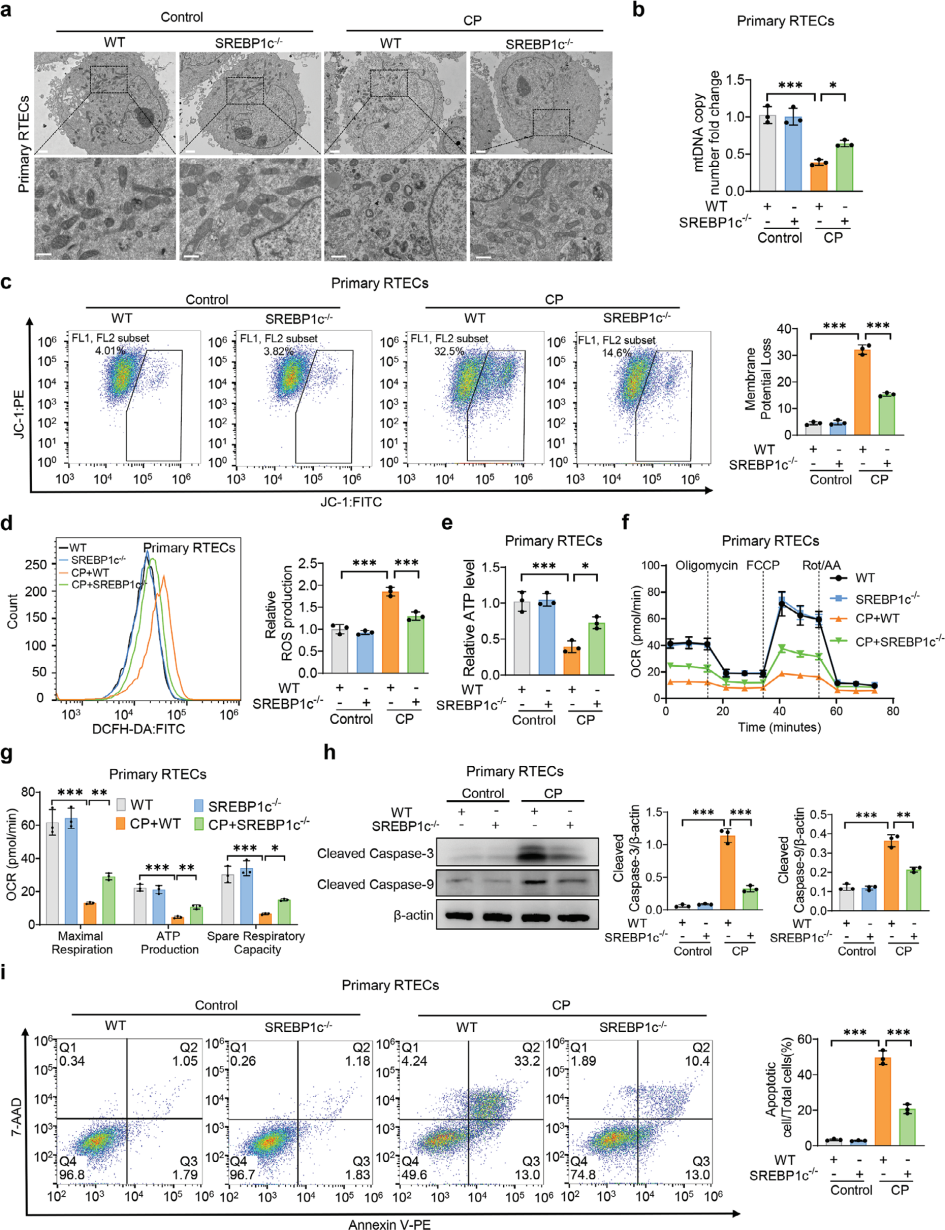
Knockout of SREBP1c alleviates cisplatin‐induced mitochondrial dysfunction in vitro. a–i) Primary RTECs from WT and SREBP1c KO mice were exposed to control or 20 µm CP for 24 h. Then Cells were harvested for TEM observation of mitochondria (a, scale bar, 2 µm (top) and 0.5 µm (bottom)), relative mtDNA copy number (b) (*n* = 3), detection of monomer and aggregate JC‐1(c) (*n* = 3), measurement of ROS production (d) (*n* = 3), relative ATP levels (e) (*n* = 3), oxygen consumption rate (OCR) (f and g) (*n* = 3), and Western blot analysis of cleaved caspase‐3 and cleaved caspase‐9 expression (h) (*n* = 3), flow cytometry analysis of apoptotic cells (i) (*n* = 3). Data are shown as means ± SD and were analyzed by one‐way ANOVA (b‐i). *P < 0.05, **P < 0.01, and ***P < 0.001.

To investigate the impact of SREBP1c on cisplatin‐induced mitochondrial dysfunction, we treated primary isolated RTECs from SREBP1c knockout (KO) mice with cisplatin. It was found that knockout of SREBP1c significantly reversed cisplatin‐induced elevation of KIM1 and NGAL expression levels and mitochondrial injury (**Figure** **5**a; Figure S7a,b, Supporting Information). Moreover, knockout of SREBP1c ameliorated cisplatin‐induced decline in mtDNA, depolarization of mitochondrial membrane potential, elevation of mitochondrial ROS production, decrease of ATP levels, repression of OCR, and suppression of FAO and OXPHO‐related genes (Figure 5b–g; Figure S7c,d, Supporting Information). Further, deficiency of SREBP1c attenuated apoptosis and balanced L‐OPA1/S‐OPA1 expression in cisplatin‐treated RTECs (Figure 5h,i; Figure S7e, Supporting Information). These results hint that knockout of SREBP1c relieves cisplatin‐induced mitochondrial dysfunction in vitro.

### 2.6 Knockout of SREBP1c Improves Cisplatin‐Induced YME1L1 Reduction, Mitochondrial Damage, AKI, and Subsequent CKD In Vivo

**Figure 6:**
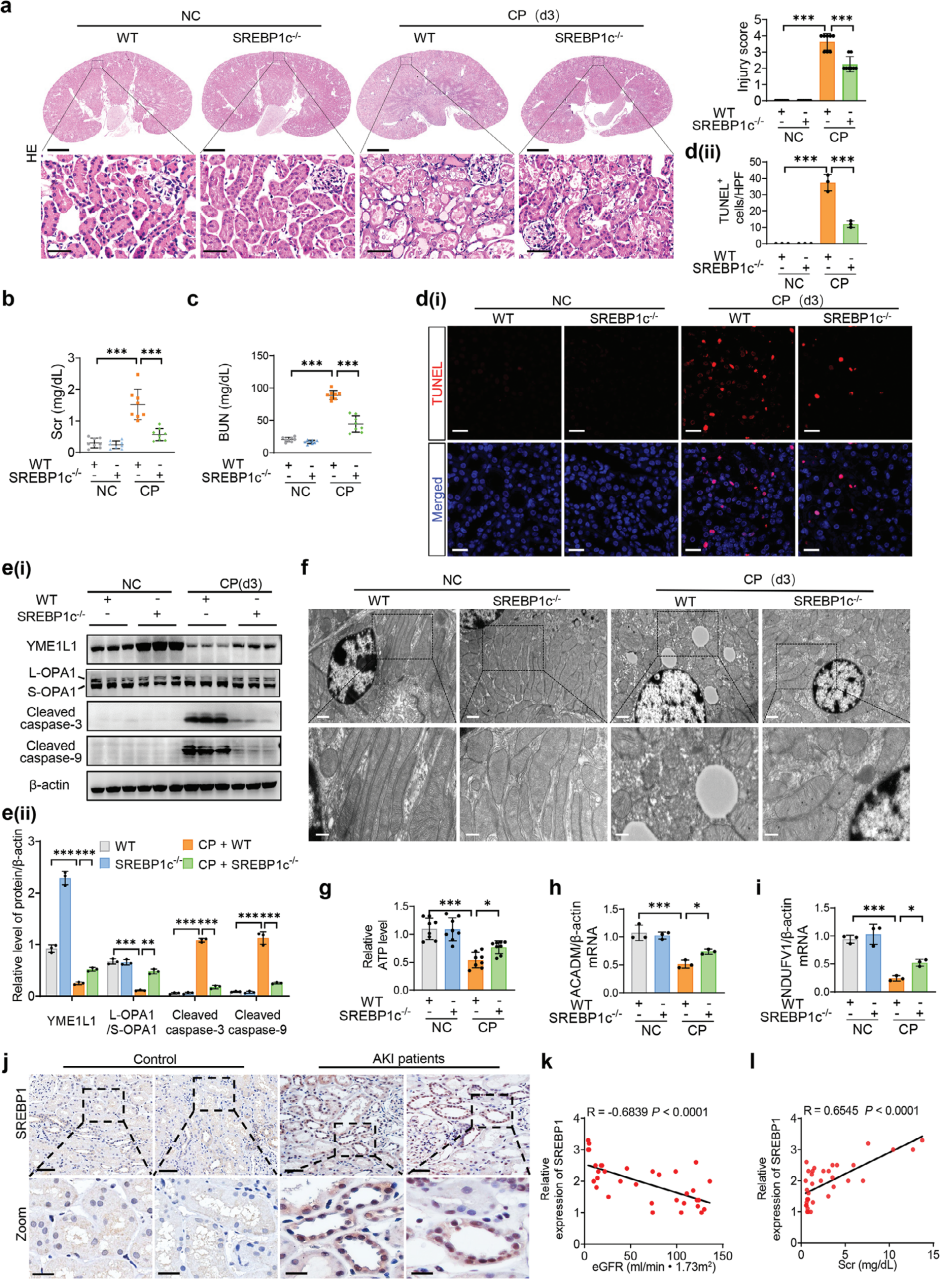
Knockout of SREBP1c improves cisplatin‐induced YME1L1 reduction, mitochondrial injury and AKI. a) WT and SREBP1c^‐/‐^ mice were injected with saline or CP (25 mg kg^−1^) and then sacrificed after 3 days for HE staining and scoring of renal tubular injury. Scale bar, 1 mm (top) and 50 µm (bottom). (*n* = 8 mice in each group). b,c) The serum from mice in (a) were collected for detecting the levels of Scr (b) and BUN (c) (*n* = 8). d–i) The kidney tissues from mice in (a) were harvested for TUNEL staining (d, scale bar, 20 µm), Western blot analysis of YME1L1, L‐OPA1/S‐OPA1, cleaved caspase‐3 and cleaved caspase‐9 expression (e) (*n* = 3), TEM observation (f, scale bar, 1 µm (top) and 0.5 µm (bottom)), determination of relative ATP levels (g) (*n* = 8), and qPCR analysis of ACADM and NDUFV1 (h and i) (*n* = 3). (j) Representative images of IHC staining of SREBP1 in the kidney biopsies from AKI patients (*n* = 21) and Para‐carcinoma tissues of renal nephrectomy samples from patients with kidney cancer (*n* = 15). Scale bar, 50 µm. k,l) Correlation analysis between SREBP1 staining intensity and renal function. Data are shown as means ± SD and were analyzed by one‐way ANOVA (a‐e and g‐i) or Spearman's rank correlation test (k and l). *P < 0.05, **P < 0.01, and ***P < 0.001.

To further demonstrate the role of SREBP1c/YME1L1 signaling in cisplatin‐induced AKI, we constructed a cisplatin‐induced AKI model using SREBP1c KO mice (Figure S8a, Supporting Information). As anticipated, SREBP1c‐deficiency not only reduced serum creatinine (Scr) and blood urea nitrogen (BUN) levels, but also released tubular damage and apoptosis in cisplatin‐induced AKI mice (**Figure** **6**a–d; Figure S8b,c, Supporting Information). Additionally, the cisplatin‐mediated downregulation of YME1L1 and L‐OPA1/S‐OPA1 expression and mitochondrial dysfunction were also restored in SREBP1c^‐/‐^ mice, as evidenced by restoration of ATP levels, upregulation of FAO and OXPHO‐related genes (Figure 6e–i).

It has been reported that maladaptive repair after AKI tends to predispose patients to the development of CKD, which is characterized primarily by renal fibrosis.^[^ ^11^ ^]^ Therefore, we extended the observation period to 28 days after cisplatin administration. Masson staining, immunofluorescence and Western blot analysis showed that cisplatin‐induced renal fibrosis was significantly attenuated in SREBP1c‐deficient mice (Figure S9a–c, Supporting Information). Notably, IHC staining demonstrated increased SREBP1 expression in AKI patients, with staining intensity inversely correlated with renal function (Figure 6j–l). Further analysis revealed a negative correlation between YME1L1 and SREBP1 expression in human kidney tissues (Figure S9d, Supporting Information). Taken together, these findings suggest that knockout of SREBP1c attenuates cisplatin‐induced YME1L1 inhibition, mitochondrial dysfunction, AKI and its progression to CKD in vivo.

### 2.7 METTL3 Regulates m6A Enrichment and Stability of SREBP1c mRNA

**Figure 7:**
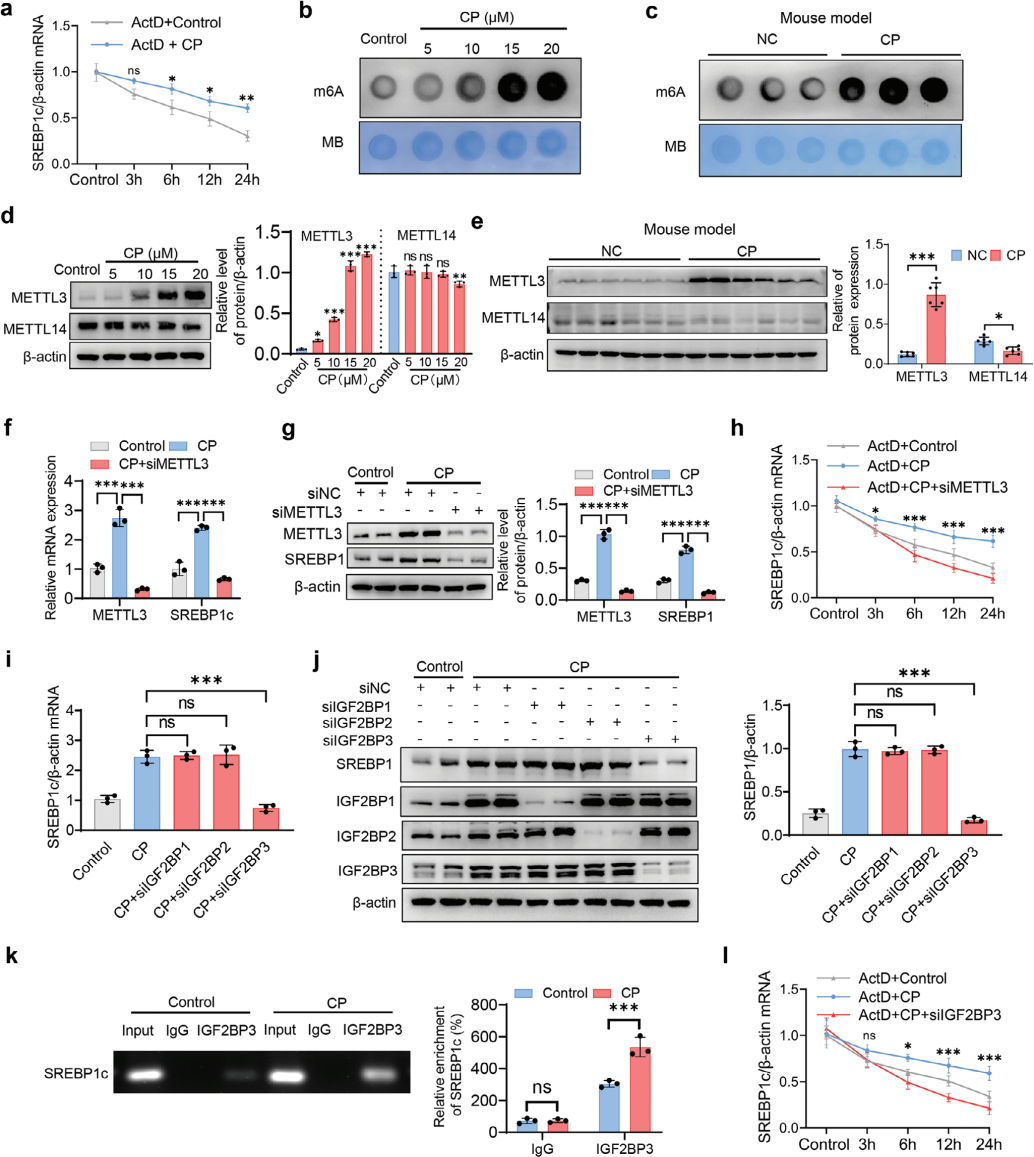
METTL3 regulates m6A enrichment and stability of SREBP1c mRNA. a) HK‐2 cells were treated with the transcriptional inhibitor actinomycin D (ActD, 0.5 µg ml^−1^) of different durations, both in the absence and presence of CP. SREBP1c mRNA expression was determined by qPCR (*n* = 3). b,c) m6A mRNA methylation were assessed via m6A dot blot in HK‐2 cells treated with different concentrations of CP for 24 h (*n* = 3) and kidney tissues from AKI mice induced by CP (25 mg kg^−1^). (*n* = 8 mice in each group). d,e) The expression levels of METTL3 and METTL14 were analyzed by Western blot in HK‐2 cells in (b) (*n* = 3) and kidney tissues in (**c**) (*n* = 8 mice in each group). f,g) HK‐2 cells were transfected with siMETTL3 or siNC and then treated with control or 20 µm CP for 24 h. Cells were collected for qPCR and Western blot analysis of METTL3 and SREBP1c expression (f and g) (*n* = 3) analysis of METTL3 and SREBP1 expression (g) (*n* = 3). (h) HK‐2 cells were transfected with siMETTL3 or siNC and treated with Actd for the indicated time in the absence or presence of CP. Cells were collected for qPCR analysis of SREBP1c expression. *P < 0.05, ***P < 0.001 versus Actd‐siMETTL3 group with CP treatment (*n* = 3). i,j) HK‐2 cells were transfected with siIGF2BP1/2/3 or siNC, and then treated with control or 20 µm CP for 24 h. Cells were collected for qPCR analysis of SREBP1c expression (i) (*n* = 3) and Western blot analysis of SREBP1 and IGF2BP1/2/3 expression (j) (*n* = 3). k) RIP assay was conducted in HK‐2 cells treated with control or CP for 24 h. Agarose electrophoresis and qPCR analysis showed the direct binding between IGF2BP3 protein and SREBP1c mRNA (*n* = 3). l) HK‐2 cells were transfected with siIGF2BP3 or siNC, and treated with Actd for the indicated time in the absence or presence of CP. Cells were collected for qPCR analysis of SREBP1c. ns: no significance, *P < 0.05, ***P < 0.001 versus Actd‐siIGF2BP3 group with CP treatment (*n* = 3). Data are shown as means ± SD and were analyzed by two‐way ANOVA (a, h, and l), two‐tailed unpaired Student's *t*‐test (e and k) or one‐way ANOVA (d, f, g, i, and j). ns: no significance. *P < 0.05, **P < 0.01, ***P < 0.001.

To explore the mechanism of cisplatin‐mediated upregulation of SREBP1c, we found that cisplatin significantly enhanced SREBP1c mRNA stability (**Figure** **7**a). Since multiple studies have indicated the crucial role of m6A in regulating mRNA stability,^[^ ^12^ ^]^ we hypothesized that m6A might be involved in cisplatin‐mediated upregulation of SREBP1c. As expected, m6A dot blot revealed that m6A RNA modifications were significantly enhanced in cisplatin‐induced AKI mice and HK‐2 cells compared to control groups (Figure 7b,c), while the expression levels of the classic methyltransferase METTL3, rather than methyltransferase‐like 14 (METTL14), were significantly upregulated (Figure 7d,e). Similarly, modification of m6A RNA was also enhanced in HK‐2 cells with HR injury and kidney tissues from IRI mice (Figure S10a–c, Supporting Information). Moreover, siRNA‐mediated knockdown of METTL3 significantly suppressed the mRNA stability and expression level of SREBP1c in HK‐2 cells exposed to HR injury or cisplatin treatment (Figure 7f–h; Figure S10d–h, Supporting Information), indicating that m6A modification of SREBP1c enhances its mRNA stability.

As reported, m6A modification requires recognition by m6A‐binding proteins, mainly insulin‐like growth factor 2 binding protein (IGF2BP) family members (IGF2BP1, 2 and 3).^[^ ^13^ ^]^ Among these reader proteins, only the knockdown of IGF2BP3 significantly decreased the mRNA and protein levels of SREBP1c in HK‐2 cells (Figure 7i,j). Subsequent RNA immunoprecipitation (RIP) analysis with an IGF2BP3 antibody further validated the interaction between IGF2BP3 and SREBP1c mRNA in HK‐2 cells treated with cisplatin (Figure 7k). Additionally, silencing IGF2BP3 also decreased the stability of SREBP1c mRNA upon cisplatin treatment (Figure 7l). These findings collectively suggest that cisplatin promotes the mRNA stability of SREBP1c by inducing METTL3‐mediated m6A modification via binding to IGF2BP3.

### 2.8 YME1L1 Overexpression Ameliorates Cisplatin‐Induced AKI and Subsequent CKD

**Figure 8:**
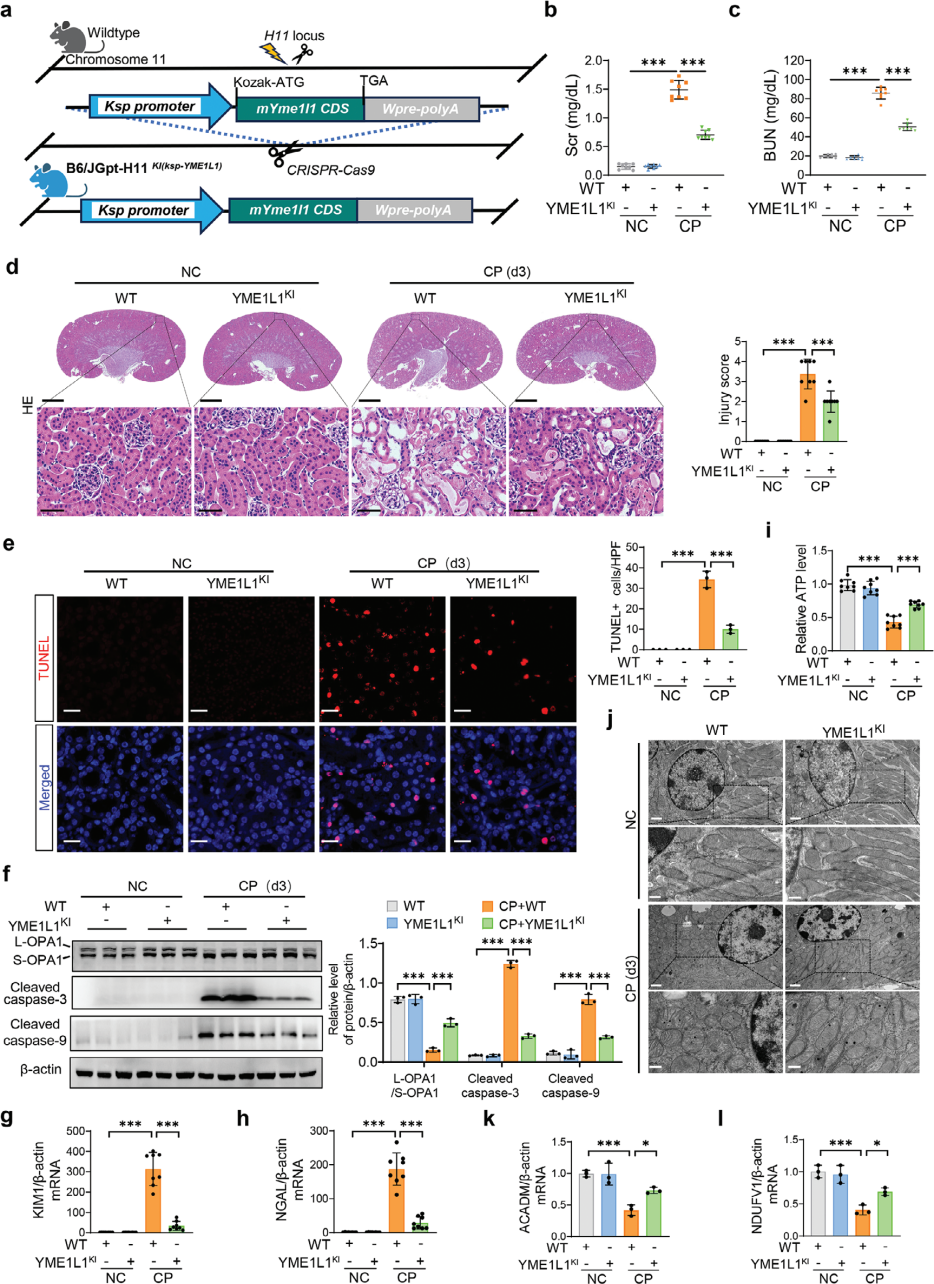
YME1L1 overexpression relieves cisplatin‐induced AKI. a) Schematic diagram for the construction of renal tubule‐specific knock‐in YME1L1 mice (YME1L1^KI^). b–l) WT or YME1L1^KI^ mice were injected with a single dose of saline or 25 mg kg^−1^ CP. (*n* = 8 mice in each group). Mice were sacrificed on day 3 for the detection of AKI indicators (Scr and BUN) (b and c) (*n* = 8), HE staining, and scoring of renal tubular injury (d, scale bar, 1 mm (top) and 50 µm (bottom)), TUNEL staining (e, scale bar, 20 µm), Western blot analysis of the expressions of L‐OPA1/S‐OPA1, cleaved caspase‐3 and cleaved caspase‐9 (f) (*n* = 3), qPCR analysis of KIM1 and NGAL (g and h) (*n* = 8), ATP levels (i) (*n* = 8), TEM observation (j, scale bar, 1 µm (top) and 0.5 µm (bottom)), and qPCR analysis of ACADM and NDUFV1 (k and l) (*n* = 3). Data are shown as means ± SD and were analyzed by one‐way ANOVA (b–i, k, and l). *P < 0.05, ***P < 0.001.

To elucidate the role of YME1L1 in vivo, mice with renal tubule‐specific overexpression of YME1L1 were generated (**Figure** **8**a; Figure S11a,b, Supporting Information). YME1L1^KI^ mice significantly rescued cisplatin‐induced renal tubular injury and renal function decline, as compared to WT mice (Figure 8b–d). Further, TUNEL staining and Western blot assays demonstrated that YME1L1 overexpression attenuated apoptosis of renal tubular cells during cisplatin administration (Figure 8e,f). Subsequently, we also observed that cisplatin‐induced damaged mitochondria, imbalanced L‐OPA1/S‐OPA1 expression, decreased ATP levels and reduced FAO and OXPHO‐related gene expression were significantly ameliorated by overexpression of YEM1L1 (Figure 8g–l). In addition, cisplatin‐induced renal interstitial fibrosis was reduced after YME1L1 overexpression (Figure S12, Supporting Information). Additionally, to explore the therapeutic role of exogenous YME1L1 in AKI, C57BL/6J mice were injected with AD‐YME1L1‐GFP (AD‐YME1L1) adenovirus (Figure S13a,b, Supporting Information). We observed that cisplatin‐induced renal tubular injury and interstitial fibrosis were also attenuated after AD‐YME1L1 treatment (Figures S13c–m and S14, Supporting Information). Taken together, these data demonstrate that both endogenous and exogenous YME1L1 alleviate AKI and subsequent chronic renal fibrosis via protecting against cisplatin‐induced mitochondrial injury.

## 3 Discussion

Mitochondrial dysfunction is recognized as a primary characteristic and key process in AKI,^[^ ^14^ ^]^ serving both as an initiator and contributor.^[^ ^4^ ^]^ In particular, mitochondrial proteins, such as Aldehyde dehydrogenase family 1 member L2 (ALDH1L2),^[^ ^15^ ^]^ Sirtuin 3 (Sirt3),^[^ ^16^ ^]^ and Sirtuin 5 (Sirt5),^[^ ^17^ ^]^ play crucial roles in maintaining mitochondrial function, thereby preventing or ameliorating AKI and its progression to CKD. Consequently, multiple studies have targeted mitochondrial dysfunction as a therapeutic approach for AKI.^[^ ^4^, ^5^, ^18^ ^]^ In this study, we screened and identified that YME1L1, a key protein in the inner mitochondrial membrane,^[^ ^19^ ^]^ downregulated in PTECs under AKI conditions both in vitro and in vivo. However, its potential roles and underlying mechanisms in AKI have not been fully explored. We found that restoration of YME1L1 expression significantly attenuated cisplatin‐induced AKI and subsequent CKD via alleviating mitochondrial energy metabolism dysfunction. Mechanistically, SREBP1c repressed cisplatin‐mediated YME1L1 transcription by binding to the YME1L1 promoter region, while METTL3‐mediated modification of m6A facilitated cisplatin‐induced upregulation of SREBP1c. Conversely, depletion of SREBP1c markedly alleviated cisplatin‐induced AKI and chronic renal fibrosis. Moreover, the expression levels of SREBP1 and YME1L1 were closely correlated with the severity of kidney damage in AKI patients. Therefore, targeting the SREBP1c/YME1L1 signaling may serve as a novel strategy for treating AKI.

Previous studies have indicated that YME1L1, an i‐AAA protease (ATPases associated with diverse cellular activities) with its protease domain anchored in the inner membrane, drives mitochondrial proteolytic rewiring and mitochondrial biogenesis mainly through degrading mitochondrial protein translocases in response to hypoxia or amino acid starvation.^[^ ^6^, ^19^ ^]^ In this study, we observed that YME1L1 expression was reduced in cisplatin‐induced HK2 cells and AKI mice, and its restoration significantly ameliorated mitochondrial structural damage in vitro and in vivo. Other studies also reported that deletion of YME1L impaired mitochondrial structure and respiratory chain biogenesis in human embryonic kidney 293 (HEK293) cells.^[^ ^20^ ^]^ These findings suggest that YME1L1 is crucial for preserving mitochondrial integrity and function. Besides, YME1L is capable of maintaining mitochondrial fusion and fission via regulating the cleavage and processing of OPA1 at S2 site.^[^ ^7^, ^21^ ^]^ In the present study, we showed that the reduction of YME1L1 disrupted this balance and exacerbated mitochondrial damage under AKI conditions. Further, we identified a novel function of YME1L1, which was able to restore mitochondrial energy metabolism homeostasis, including upregulation of the expression of OXPHO and FAO‐related genes, reduction of ROS accumulation, and increase of ATP production, thereby effectively alleviating cisplatin‐induced mitochondrial dysfunction and apoptosis in vitro and in vivo. These findings demonstrate the vital role of YME1L1 in maintaining mitochondrial structure and energy metabolism homeostasis in RTECs.

As a small‐molecule platinum compound, cisplatin cannot directly inhibit the transcription of the YME1L1 gene. Thus, we sought to explore the mediators, and bioinformatic screening coupled with experimental validations identified SREBP1c as the transcription factor mediating the cisplatin‐induced downregulation of YME1L1 in PTECs. Normally, SREBP1c, part of the SREBP family, is embedded in the endoplasmic reticulum as hairpins.^[^ ^22^ ^]^ Under high glucose‐induced diabetes,^[^ ^23^ ^]^ obesity‐associated nephropathy,^[^ ^24^ ^]^ and other tissue‐injury stimuli,^[^ ^22^ ^]^ SREBP1c undergoes proteolytic cleavage to produce a mature protein that enters the nucleus and modulates the transcription of target genes by binding to their sterol regulatory elements (SREs).^[^ ^25^ ^]^ Here, we identified YME1L1 as a novel target gene of SREBP1c, which represses its expression by directly binding to the promoter region (‐214 to ‐205, 5′‐ATCGCGCCAC‐3′). This negative association between YME1L1 and SREBP1c was further confirmed in human kidney tissues from control and AKI patients. It was also demonstrated that the ablation of SREBP1c could mitigate cisplatin‐induced YME1L1 downregulation, mitochondrial dysfunction, and AKI. This study is the first to identify the YME1L1 as a target of SREBP1c, elucidating a SREBP1c/YME1L1 axis in response to cisplatin‐induced maladaptive repair of renal tubules in AKI, thus offering novel insights into the pathogenesis of AKI.

Cumulative studies have shown that, AKI triggers reprogramming of energy metabolism, which in turn aggravates organ dysfunction, chronic fibrosis, and disease progression.^[^ ^26^ ^]^ In AKI, the primary mode of energy metabolism in renal PTECs shifts from FAO to glycolysis.^[^ ^27^ ^]^ Targeting energy metabolism in PTECs could be a potential strategy for altering renal metabolic reprogramming and promoting kidney repair.^[^ ^28^ ^]^ Of note, SREBP1c acts as a key factor in metabolic regulation.^[^ ^29^ ^]^ Previous studies found that SREBP1 was activated in AKI to induce cholesterol load,^[^ ^30^ ^]^ but the mechanisms by which it regulates energy metabolism in AKI remain to be clarified. This study revealed that suppression of SREBP1c significantly reverses cisplatin‐induced mitochondrial energy metabolism remodeling and mitigates AKI. Although we isolated primary RTECs from SREBP1c KO mice to validate this effect, future studies utilizing RTECs‐specific SREBP1c knockout mouse model may further demonstrate the specific role of SREBP1c in AKI.

As reported, mitochondrial damage is an early event in AKI, usually within several hours following AKI onset, before the occurrence of lipid metabolic disorders.^[^ ^5^, ^31^ ^]^ This study indicated that the expression of SREBP1c significantly increased as early as 6 h after cisplatin treatment, accompanied by the suppression of YME1L1 transcription. These results suggest that SREBP1c may induce mitochondrial dysfunction by downregulating YME1L1 in the early phase of AKI. Besides, multiple studies have shown that SREBP1c‐regulated lipid metabolism disorders usually occur in chronic metabolic diseases.^[^ ^32^ ^]^ It can upregulate classical lipogenic genes, such as FASN and ACC1, contributing to lipid homeostasis imbalance in diabetic nephropathy,^[^ ^10^, ^33^ ^]^ as well as enhancing lipotoxicity in obesity‐related nephropathy.^[^ ^34^ ^]^ Therefore, in cisplatin‐induced AKI mouse model, we also examined the expression of lipogenic genes, FASN and ACC1, at different time periods, and found that they were only mildly elevated or even unchanged during the pre‐AKI phase until day 4, indicating that SREBP1c‐regulated lipid metabolic disorders may not exacerbate mitochondrial damage in the early stages of AKI. Nevertheless, we still cannot exclude its role in the late stages of kidney injury.

The upstream regulatory network of SREBP1c remains largely unexplored. This study discovered that METTL3 enhances SREBP1c levels by increasing the stability of its mRNA through m6A modification. m6A modification, a prevalent RNA modification in eukaryotic mRNA, is crucial for gene expression regulation.^[^ ^12^, ^35^ ^]^ For example, in sepsis‐associated lung injury, METTL3‐mediated m6A modification enhanced the stability of ACSL4 mRNA, thereby promoting mitochondrial‐associated ferroptosis.^[^ ^36^ ^]^ However, the role of METTL3 in renal mitochondria, particularly in mitochondria‐rich RTECs, has not been identified. We demonstrated that METTL3 is markedly induced in AKI, and its mediated m6A modification increases SREBP1c mRNA stability through binding to IGF2BP3, leading to mitochondrial energy metabolism dysfunction in RTECs.

Interestingly, a strong correlation was observed between the expression levels of SREBP1c and YME1L1 and the severity of AKI in both control and AKI patients, suggesting the SREBP1c/YME1L1 signaling as a promising biomarker for AKI. However, further validations are needed through expanded sample sizes and multicenter randomized clinical trials.

## 4 Conclusion

In conclusion, the present study reveals YME1L1‐mediated modulation of mitochondrial energy metabolism, identifies the downregulation of YME1L1 as a contributor to AKI, and illuminates the underlying mechanism that transcriptional factor SREBP1c mediates cisplatin‐induced suppression of YME1L1 via direct combining with its promoter domain. In this process METTL3 is crucial for the upregulation of SREBP1c mediated by m6A modification. Both overexpression of YME1L1 and knockout of SREBP1c ameliorates mitochondrial energy metabolism dysfunction in renal PTECs, thereby retarding the progression of AKI and subsequent CKD. These findings collectively suggest SREBP1c/YME1L1 signaling as a novel therapeutic target for the treatment of AKI.

## 5 Experimental Section

### Patient Samples Collection

Twenty‐one patients with AKI and acute tubular necrosis verified by renal biopsy from Xinqiao Hospital of Army Medical University were enrolled. Fifteen para‐carcinoma tissues of renal nephrectomy samples from patients with kidney cancer were used as control (Table S1, Supporting Information). Informed consent was obtained from all participants, and all procedures involving human subjects were approved by the Ethics Committee of Xinqiao Hospital, Army Medical University, following the Declaration of Helsinki guidelines.

### Mouse Models

SREBP1c^‐/‐^ mice (C57BL/6J background) were obtained from Jackson Laboratory (B6; 129S6‐*Srebf1^tm1jdh^*, stock number: 0 04365 Bar Harbor, ME, USA) and littermate wild‐type (WT) mice served as controls. The YME1L1 kidney tubule‐specific knock‐in mice (YME1L1^KI^) were obtained from GemPharmatech (Nanjing, China), with littermate WT mice used as controls. As previously described, the *Yme1l1‐Wpre‐PolyA* gene fragment carrying the cadherin 16 (Cdh16) promoter was inserted into the Hipp11 (H11) locus of C57BL/6J mice using CRISPR‐Cas9 gene editing technology.^[^ ^37^ ^]^ F0 positive mice were crossed with C57BL/6J mice to create stably inherited heterozygous positive mice, which were further bred to produce homozygous positive mice with renal tubule specific knock‐in of YME1L1. C57BL/6J mice were purchased from Huafu Kang Biotechnology (Beijing, China). To construct exogenous YME1L1 overexpression mice, 50 µL of adenovirus expressing only GFP (mock control) or YME1L1‐GFP (≈10^11^ plaque‐forming units mL^−1^, Baiouni Biotechnology, Chongqing, China) was injected into the tail vein of mice as previously described.^[^ ^38^ ^]^

Cisplatin‐induced AKI models were established as previously reported.^[^ ^9d^ ^]^ In brief, 8‐week‐old male WT C57BL/6J, YME1L1^KI^ and SREBP1c^‐/‐^ mice were injected intraperitoneally with either saline or 25 mg kg^−1^ cisplatin (MCE, Monmouth Junction, NJ, USA). Mice were euthanized after 3 days, and blood and kidney specimens were collected. IRI‐induced AKI mouse model was created by performing a 30 min clamping of bilateral renal arteries to induce ischemia, followed by 24 h of reperfusion.^[^ ^9c^ ^]^ FA‐induced AKI mouse model was established by intraperitoneal injection of 250 mg kg^−1^ FA or vehicle, followed by euthanasia 24 h later.^[^ ^9d^ ^]^ The AKI‐to‐CKD mouse model was established as previously reported,^[^ ^39^ ^]^ with two injections of cisplatin (15 mg kg^−1^) on days 0 and 14, followed by euthanasia and collection of blood and kidney specimens on day 28. All animal procedures were approved by the Animal Experimentation Ethics Committee at Army Medical University (No. AMUWEC20224523).

### Cell Culture and Treatment

HK‐2 cells were purchased from the American Tissue Culture Collection (ATCC, Manassas, VA, USA) and maintained in DMEM/F12 medium (Meilunbio, Dalian, China) containing 10% fetal bovine serum (FBS, Corning, Corning, NY, USA) at 37 °C in a humidified environment. To construct HR model of HK‐2 cells, the cells were placed in 94% N_2_, 1% O_2_, and 5% CO_2_ atmosphere with glucose and serum‐free DMEM/F12 medium hypoxia treatment for 24 h. Subsequently, DMEM/F12 medium containing glucose and 10% FBS was replaced, and reoxygenation was continued in 95% air and 5% CO_2_, and cells were collected at 2, 4, and 6 h for analysis. To construct a cisplatin‐induced HK‐2 cell model, HK‐2 cells were exposed to varying concentrations of cisplatin for 24 h, after which the cells were collected for subsequent analysis.

### Cell Transfection

HK‐2 cells were transfected with YME1L1 or SREBP1c overexpression plasmids (Youbio, Hunan, China) or siRNA (YME1L1, OPA1, METTL3, IGF2BP1, IGF2BP2, IGF2BP3) using Lipofectamine 3000 (Invitrogen, Carlsbad, CA, USA). Cells were transfected for 24 h, and then treated with cisplatin for 24 h. The siRNAs targeting YME1L1, OPA1, METTL3, IGF2BP1, IGF2BP2, and IGF2BP3 (listed in Table S6, Supporting Information) were synthesized by Biomics (Jiangsu, China). The knockout of EGR1, FOSB, and SREBP1c in HK‐2 cells was achieved through CRISPR/Cas9 genome editing.^[^ ^40^ ^]^ The sgRNA sequences (listed in Table S7, Supporting Information) were inserted into the pRPCas9(BB)‐2A‐Puro vector. Then the plasmids containing the sgRNA (VectorBuilder, Guangzhou, China) were transfected into HK‐2 cells. After 48 h, the cells were screened with puromycin (1 µg mL^−1^). Knockdown efficiency was confirmed by Western blot.

### Mouse Primary Tubule Isolation and Cell Culture

Mouse primary renal tubule cells culture was performed as previously described.^[^ ^41^ ^]^ Briefly, WT C57BL/6J mice and SREBP1c^‐/‐^ mice (4 weeks old) were used for primary renal tubule isolation. The kidneys were harvested, and the renal cortex was minced and digested for 8 min via 0.1% collagenase II (Sigma‐Aldrich, St. Louis, MO, USA). The resulting tissue homogenate was then filtered twice through a 70 µm filter (BD Falcon, Franklin Lakes, NJ, USA). The samples were collected and centrifuged at 1000 g to obtain pellets containing renal tubules. Primary renal tubule cells were placed in DMEM/F12 medium containing 10% FBS.

### Flow Assay for Mouse Primary RTECs

Primary renal tubular cells were extracted from the renal cortex of both control and cisplatin‐induced AKI mice. These cells were then labeled with Lotus tetragonolobus lectin (LTL), a marker for proximal tubular epithelial cells. Subsequently, cells were fixed and stained with primary antibody against YME1L1 (PA5‐24808, Invitrogen) and PE‐conjugated secondary antibody. The cells were analyzed with a Gallios flow cytometer (Beckman Coulter, Brea, CA, USA).

### qRT‐PCR

Total RNA was extracted using Trizol reagent (T9424, Sigma‐Aldrich), followed by reverse transcription using the reverse transcription kit (MCE, Monmouth Junction, NJ, IUSA) and qPCR analysis using a SYBR Green qPCR kit (MCE, Monmouth Junction, NJ, USA). Total DNA from RTECs and HK‐2 cells was extracted using a DNA extraction kit (Takara, Shiga, Japan) as previously described,^[^ ^42^ ^]^ and mtDNA copy number was analyzed using qPCR. Primers for human and mouse are provided in Tables S4 and S5 (Supporting Information).

### Western Blot

Total proteins were lysed with Ripa Lysis Buffer (Beyotime, Shanghai, China). Western blot was performed using primary antibodies against YME1L1 (11510‐1‐AP), Mfn1 (13798‐1‐AP), Fis1 (10956‐1‐AP), METTL14 (26158‐1‐AP), IGF2BP1 (22803‐1‐AP), METTL3 (15073‐1‐AP), IGF2BP2 (11601‐1‐AP), IGF2BP3 (14642‐1‐AP), EGR1 (22008‐1‐AP) from Proteintech (Wuhan, China); Mfn2 (ab124733), OPA1 (ab90857), Drp1 (ab56788), SREBP1 (ab28481), Fibronectin (ab268020) from Abcam (MA, USA); FOSB (sc‐398595) from Santa Cruz; α‐SMA (48938S), Cleaved caspase‐3 (9664S), Cleaved caspase‐9 (20750S) from CST (Danvers, MA, USA) or β‐actin (AF0003) from Beyotime as described previously.^[^ ^43^ ^]^

### Dot Blot

Total RNA was isolated with TRIzol. The RNA concentration was adjusted to 100 ng µL^−1^, and 2 µL of the quantified RNA solution was applied to a nylon membrane. The membranes were cross‐linked under UV light and blocked with PBS‐T containing 5% BSA. Subsequently, the membrane was incubated overnight using m6A antibody (ab284130, Abcam). After that, it was incubated with the secondary antibody, and the signal was detected using ECL chemiluminescence reagents (17 047, Zenbio, Chengdu, China). Finally, the total RNA level was assessed by methylene blue staining at 0.02%.

### ATP Measurement

Cells or mouse kidney tissues were lysed and centrifuged, and ATP levels were quantified using the ATP Assay Kit (S0026, Beyotime) following the provided instructions. ATP concentrations were calculated based on the standard curve, and protein content was measured to standardize ATP levels (nmol) per milligram of total protein. The relative ATP levels in each sample were then analyzed in comparison to the control group.

### Cell Mito Stress Test

The OCR was measured by using the Seahorse XF HS Mini Analyzer (Agilent Technologies, Santa Clara, CA, USA). HK‐2 cells or primary RTECs were plated in XF8 culture plates (13 022, Agilent Technologies) and treated with cisplatin (20 µm) for 24 h once they reached a density of 2 × 10⁴ cells per well. The Seahorse XFp Cell Mito Stress Test Kit (103 010, Agilent Technologies) was employed to measure OCR. Following three baseline respiration readings without any additions, 1.5 µm oligomycin was introduced, followed by the addition of 2 µm FCCP and Rotenone & antimycin A (0.5 µm Rot/AA) for further measurements. OCR was expressed as units of picomoles (pmol) per minute, and data were normalized against the number of cells.

### Mitochondrial Membrane Potential and ROS Assay

HK‐2 cells or primary RTECs were treated with JC‐1 (C2003S, Beyotime) or the fluorescent probe DCFH‐DA (S0033M, Beyotime), and subsequently analyzed by using a flow cytometer.

### Apoptosis Assay

Apoptosis analysis was performed following the manufacturer's guidelines (559 763, BD biosciences). HK‐2 cells or primary RTECs were incubated with the fluorescent probe Annexin V and 7‐AAD for 20 min and subsequently analyzed by using a flow cytometer. TUNEL staining was conducted with a TUNEL assay kit (Roche, Mannheim, Germany) and visualized using confocal microscopy (Zeiss, Oberkochen, Germany).

### Detection of Scr and BUN

Mouse Scr and BUN levels were measured using a creatinine assay kit (C011‐2; njjcbio, China) and a urea nitrogen measurement kit (C013‐2; njjcbio), respectively, according to the protocol specified by the manufacturer.

### Transmission Electron Microscopy

HK‐2 cells, primary RTECs, and mouse renal tissues were fixed in glutaraldehyde and 2% osmium tetroxide for 1 h at 37 °C, then dehydrated, embedded, sectioned ultrathin, and visualized using TEM (JEM‐1400PLUS, Japan).^[^ ^42^ ^]^

### HE and Masson Staining

The kidney tissues were sectioned to a thickness of 3 µm and stained with hematoxylin and eosin, as well as Masson trichrome, for light microscopic analysis.

### Immunofluorescence

Primary RTECs were fixed with 4% paraformaldehyde, then blocked in 2% BSA. Cells were incubated overnight with primary antibody against YME1L1 (ab234744, Abcam), followed by an FITC‐conjugated secondary antibody. Kidney tissue cryosections (4 µm thick) underwent similar fixation, permeabilization, blocking, and overnight incubation with primary antibodies against YME1L1 (11510‐1‐AP, Proteintech), Fibronectin (ab268020, Abcam), and α‐SMA (48938S, CST), followed by secondary antibodies and DAPI. Visualization was performed using a confocal microscope.

### Immunohistochemistry

Kidney biopsy tissues from AKI patients and control sections underwent antigen repair and were incubated with anti‐SREBP1 (ab28481, Abcam) or anti‐YME1L1 (ab234744, Abcam) antibodies. Visualization was performed using a DAB kit (ZSGB‐BIO, Beijing, China). As previously stated,^[^ ^44^ ^]^ tubular staining was scored by two independent researchers on a scale of 0‐4: score 0 indicated no specific staining; score 1 represented less than 25% of the area with specific staining; score 2 indicated 25% to 50%; score 3 corresponded to 50% to 75%; and score 4 indicated more than 75% of the area.

### Construction of Reporter Plasmids

According to the predicted binding sites of SREBP1 in the YME1L1 promoter region, corresponding primers (Table S8, Supporting Information)) were designed to amplify different lengths of the YME1L1 promoter. The fragments including YME1L1‐2000 (−2000 to +200), YME1L1‐1000 (−1000 to +200), YME1L1‐700 (−700 to +200), YME1L1‐400 (−400 to +200), and YME1L1‐150 (−150 to +200) were individually inserted into pGL3 basic vector following digestion with KpnI and XhoI. The recombinant reporter plasmids were designated as pGL3‐YME1L1‐P1 through pGL3‐YME1L1‐P5, respectively. Point mutations were introduced into the SREBP1 binding element of pGL3‐YME1L1‐M3 (CCGAGAACCCGACGCTGTAC, Underlines represent mutated bases) with pGL3‐YME1L1‐P4 (−400 to +200) serving as the positive control.

### Dual Luciferase Reporter Analysis

Recombinant reporter plasmids and subsequent point mutation plasmids were constructed based on the promoter region of YME1L1. The recombinant plasmids and Renilla plasmids, along with the pGL3‐basic vector, were co‐transfected into HK‐2 cells alongside either the pCDNA3.1 vector or an SREBP1c overexpression plasmid. Luciferase activity was measured using a dual luciferase reporter assay kit (Promega, Madison, WI, USA), and it was normalized using fluorescence activity with Renilla activity.

### Chromatin Immunoprecipitation (ChIP)

ChIP assays were conducted using kits from Invitrogen.^[^ ^42^ ^]^ HK‐2 cells treated with cisplatin or control for 24 h were cross‐linked with 1% formaldehyde, lysed, and sonicated to fragment DNA. Immunoprecipitation was performed with 2 µg of SREBP1 antibody (14088‐1‐AP, Proteintech). The isolated DNA was then subjected to PCR and qPCR amplification with primers designed to encompass the SREBP1 binding sites (−214 to −205). Additionally, primers targeting regions without SREBP1 binding sites were used as a negative control, while total DNA (Input) acted as a positive control (The primers are listed in Table S9, Supporting Information).

### RNA Immunoprecipitation

The RIP assay was assessed via a kit from Millipore (Burlington, MA, USA) following the manufacturer's guidelines.^[^ ^45^ ^]^ HK‐2 cells were exposed to either control conditions or cisplatin for 24 h. Cells were lysed by adding 150 µl RIP lysis buffer. Subsequently, 5 µg of anti‐mouse beads containing antibodies specific for IgG or IGF2BP3 (14642‐1‐AP, Proteintech) were incubated for 6 h. Next, the RNA‐protein complexes were treated with proteinase K to extract the RNA. SREBP1c mRNA was assayed using qPCR to quantify its interaction with IGF2BP3.

### Bioinformatics Analysis

The microarray data set (GSE87025)^[^ ^46^ ^]^ was retrieved from the Gene Expression Omnibus (GEO) database. Differential expression analysis was then conducted using limma package (version 3.50.3).^[^ ^47^ ^]^ Genes were considered differentially expressed if they met the criteria of |fold change| > 1 and an adjusted p‐value (FDR < 0.01). The JASPAR database (http://jaspar.genereg.net) was used to predict the binding site of SREBP1 in the *YME1L1* promoter region.^[^ ^42^ ^]^ GO analysis was performed by Metascape^[^ ^48^ ^]^ (http://metascape.org) and clusterprofiler. Mouse kidney single‐cell RNA sequencing database (GSE197266) were obtained from the NCBI GEO database.^[^ ^49^ ^]^ Analysis was conducted using the R package Seurat (version 3.1.1). Quality control for the dataset were as follows: gene counts between 500 and 4000, NUMI counts greater than 500 and less than 15000; exclusion of cells with high complexity (log10GenesPerUMI) ≤ 0.8; and removal of cells with a mitochondrial ratio exceeding 10%. Harmoniously integrate matrices for all samples and eliminate batch effects between different samples.^[^ ^50^ ^]^

### Statistical Analysis

Data were presented as mean ± SD. One‐way analysis of variance (ANOVA) was used for comparisons among multiple groups, while two‐tailed unpaired Student's *t*‐test was employed for two‐group comparisons. The correlation was examined by Spearman's rank correlation test. Statistical analysis was performed using GraphPad Prism 8.0 (GraphPad Software Inc, La Jolla, CA, USA). *P *< 0.05 was considered statistically significant.

## Conflict of Interest

The authors declare no conflict of interest.

## Author Contributions

W.X. and J.Z. contributed equally to this work. J.Z. and Y.H. designed the study and revised the manuscript. W.X. and J.Z. performed the experiments and drafted the manuscript. Y.P., S.G., W.L., X.H., Y.M., J.X., Y.L., and Q.L. analyzed the data. Y.W., M.Y., and S.Q. carried out animal studies. All authors approved the final version of the paper.

## Supporting information



Supporting Information

## Data Availability

The data that support the findings of this study are openly available in Gene Expression Omnibus database at https://www.ncbi.nlm.nih.gov/geo/, reference number 4649. These data were derived from the following resources available in the public domain: [GSE87025], https://www.ncbi.nlm.nih.gov/geo/query/acc.cgi?acc=GSE87025; [GSE197266], https://www.ncbi.nlm.nih.gov/geo/query/acc.cgi?acc=GSE197266.

## References

[1] a) R. Bellomo , J. A. Kellum , C. Ronco , Lancet 2012, 380, 756; 22617274 10.1016/S0140-6736(11)61454-2

[2] Y. Hu , C. Yang , T. Amorim , M. Maqbool , J. Lin , C. Li , C. Fang , L. Xue , A. Kwart , H. Fang , M. Yin , A. J. Janocha , D. Tsuchimoto , Y. Nakabeppu , X. Jiang , A. Mejia‐Garcia , F. Anwer , J. Khouri , X. Qi , Q. Y. Zheng , J. S. Yu , S. Yan , T. LaFramboise , K. C. Anderson , L. C. Herlitz , N. C. Munshi , J. Lin , J. Zhao , Cancer Res. 2021, 81, 713. 33288657 10.1158/0008-5472.CAN-20-1010 PMC7869671

[3] a) L. Xu , J. Guo , D. G. Moledina , L. G. Cantley , Nat. Commun. 2022, 13, 4892; 35986026 10.1038/s41467-022-32634-0 PMC9391331

[4] P. Bhargava , R. G. Schnellmann , Nat. Rev. Nephrol. 2017, 13, 629. 28804120 10.1038/nrneph.2017.107 PMC5965678

[5] a) C. Tang , J. Cai , X. M. Yin , J. M. Weinberg , M. A. Venkatachalam , Z. Dong , Nat. Rev. Nephrol. 2021, 17, 299; 33235391 10.1038/s41581-020-00369-0 PMC8958893

[6] Y. Ruan , H. Li , K. Zhang , F. Jian , J. Tang , Z. Song , Cell Death Dis. 2013, 4, 896. 10.1038/cddis.2013.414 PMC3920928 24176854

[7] Y. J. Lee , G. H. Kim , S. I. Park , J. H. Lim , J. Cell. Mol. Med. 2020, 24, 899. 31725201 10.1111/jcmm.14799 PMC6933342

[8] K. Ando , T. Yokochi , A. Mukai , G. Wei , Y. Li , S. Kramer , T. Ozaki , Y. Maehara , A. Nakagawara , Mol. Carcinog. 2019, 58, 1134. 30859632 10.1002/mc.22997 PMC6593999

[9] a) V. Volarevic , B. Djokovic , M. G. Jankovic , C. R. Harrell , C. Fellabaum , V. Djonov , N. Arsenijevic , J. Biomed. Sci. 2019, 26, 25; 30866950 10.1186/s12929-019-0518-9 PMC6417243

[10] Y. Wang , J. Viscarra , S. J. Kim , H. S. Sul , Nat. Rev. Mol. Cell Biol. 2015, 16, 678. 26490400 10.1038/nrm4074 PMC4884795

[11] L. He , Q. Wei , J. Liu , M. Yi , Y. Liu , H. Liu , L. Sun , Y. Peng , F. Liu , M. A. Venkatachalam , Z. Dong , Kidney Int. 2017, 92, 1071. 28890325 10.1016/j.kint.2017.06.030 PMC5683166

[12] a) J. S. Guo , J. Ma , X. H. Zhao , J. F. Zhang , K. L. Liu , L. T. Li , Y. X. Qin , F. H. Meng , L. Y. Jian , Y. H. Yang , X. Y. Li , Adv. Sci. (Weinh) 2024, 11, 2402450; 38952061 10.1002/advs.202402450 PMC11434010

[13] Z. Ni , P. Sun , J. Zheng , M. Wu , C. Yang , M. Cheng , M. Yin , C. Cui , G. Wang , L. Yuan , Q. Gao , Y. Li , Cancer Res. 2022, 82, 1789. 35502544 10.1158/0008-5472.CAN-21-1323

[14] A. Zuk , J. V. Bonventre , Annu. Rev. Med. 2016, 67, 293. 26768243 10.1146/annurev-med-050214-013407 PMC4845743

[15] Y. Yang , S. Liu , H. Gao , P. Wang , Y. Zhang , A. Zhang , Z. Jia , S. Huang , Free Radical Biol. Med. 2020, 152, 821. 32004633 10.1016/j.freeradbiomed.2020.01.182

[16] S. J. Allison , Nat. Rev. Nephrol. 2015, 11, 197. 10.1038/nrneph.2015.13 25668000

[17] T. Chiba , K. D. Peasley , K. R. Cargill , K. V. Maringer , S. S. Bharathi , E. Mukherjee , Y. Zhang , A. Holtz , N. Basisty , S. D. Yagobian , B. Schilling , E. S. Goetzman , S. Sims‐Lucas , J. Am. Soc. Nephrol. 2019, 30, 2384. 31575700 10.1681/ASN.2019020163 PMC6900790

[18] A. V. Birk , S. Liu , Y. Soong , W. Mills , P. Singh , J. D. Warren , S. V. Seshan , J. D. Pardee , H. H. Szeto , J. Am. Soc. Nephrol. 2013, 24, 1250. 23813215 10.1681/ASN.2012121216 PMC3736700

[19] T. MacVicar , Y. Ohba , H. Nolte , F. C. Mayer , T. Tatsuta , H. G. Sprenger , B. Lindner , Y. Zhao , J. Li , C. Bruns , M. Krüger , M. Habich , J. Riemer , R. Schwarzer , M. Pasparakis , S. Henschke , J. C. Brüning , N. Zamboni , T. Langer , Nature 2019, 575, 361. 31695197 10.1038/s41586-019-1738-6

[20] J. Cesnekova , M. Rodinova , H. Hansikova , J. Zeman , L. Stiburek , Int. J. Mol. Sci. 2018, 19, 3930. 30544562 10.3390/ijms19123930 PMC6321463

[21] a) D. C. Chan , Annu. Rev. Pathol. 2020, 15, 235; 31585519 10.1146/annurev-pathmechdis-012419-032711

[22] D. Dorotea , D. Koya , H. Ha , Front. Pharmacol. 2020, 11, 265. 32256356 10.3389/fphar.2020.00265 PMC7092724

[23] X. Wen , Y. Zeng , L. Liu , H. Zhang , W. Xu , N. Li , X. Jia , J. Ethnopharmacol. 2012, 142, 144. 22564814 10.1016/j.jep.2012.04.028

[24] D. Wang , Y. Luo , X. Wang , D. J. Orlicky , K. Myakala , P. Yang , M. Levi , Int. J. Mol. Sci. 2018, 19, 137. 29301371 10.3390/ijms19010137 PMC5796086

[25] a) A. Chen , X. Chen , S. Cheng , L. Shu , M. Yan , L. Yao , B. Wang , S. Huang , L. Zhou , Z. Yang , G. Liu , Biochim. Biophys. Acta Mol. Cell Biol. Lipids 2018, 1863, 538; 29486327 10.1016/j.bbalip.2018.02.003

[26] C. Tan , J. Gu , T. Li , H. Chen , K. Liu , M. Liu , H. Zhang , X. Xiao , Int. J. Mol. Med. 2021, 47, 19. 33448325 10.3892/ijmm.2021.4852 PMC7849980

[27] R. Lan , H. Geng , P. K. Singha , P. Saikumar , E. P. Bottinger , J. M. Weinberg , M. A. Venkatachalam , J. Am. Soc. Nephrol. 2016, 27, 3356. 27000065 10.1681/ASN.2015020177 PMC5084876

[28] S. van der Rijt , J. C. Leemans , S. Florquin , R. H. Houtkooper , A. Tammaro , Nat. Rev. Nephrol. 2022, 18, 588. 35798902 10.1038/s41581-022-00592-x

[29] H. Shimano , R. Sato , Nat. Rev. Endocrinol. 2017, 13, 710. 28849786 10.1038/nrendo.2017.91

[30] M. Naito , K. Bomsztyk , R. A. Zager , Am. J. Pathol. 2009, 174, 54. 19095962 10.2353/ajpath.2009.080602 PMC2631318

[31] a) Z. Song , Y. Xia , L. Shi , H. Zha , J. Huang , X. Xiang , H. Li , H. Huang , R. Yue , H. Wang , J. Zhu , Cell. Mol. Biol. Lett. 2024, 29, 31; 38439028 10.1186/s11658-024-00553-1 PMC10910703

[32] a) W. Zhu , M. Chen , Y. Wang , Y. Chen , Y. Zhang , Y. Wang , P. Liu , P. Li , Chem. Biol. Interact. 2023, 385, 110711; 37769864 10.1016/j.cbi.2023.110711

[33] L. Sun , N. Halaihel , W. Zhang , T. Rogers , M. Levi , J. Biol. Chem. 2002, 277, 18919. 11875060 10.1074/jbc.M110650200

[34] Y. C. Lin , M. S. Wu , Y. F. Lin , C. R. Chen , C. Y. Chen , C. J. Chen , C. C. Shen , K. C. Chen , C. C. Peng , Int. J. Mol. Sci. 2019, 20, 1570.30934807

[35] S. Oerum , V. Meynier , M. Catala , C. Tisne , Nucleic Acids Res. 2021, 49, 7239. 34023900 10.1093/nar/gkab378 PMC8287941

[36] D. Wu , C. B. Spencer , L. Ortoga , H. Zhang , C. Miao , Redox Biol. 2024, 74, 103194. 38852200 10.1016/j.redox.2024.103194 PMC11219935

[37] Z. Zhang , L. Zhou , Q. Liu , Y. Zheng , X. Tan , Z. Huang , M. Guo , X. Wang , X. Chen , S. Liang , W. Li , K. Song , K. Yan , J. Li , Q. Li , Y. Zhang , S. Yang , Z. Cai , M. Dai , Q. Xian , Z. L. Shi , K. Xu , K. Lan , Y. Chen , Emerging Microbes Infect. 2024, 13, 2353302. 10.1080/22221751.2024.2353302 PMC11132709 38753462

[38] Y. Pan , S. Jiang , Q. Hou , D. Qiu , J. Shi , L. Wang , Z. Chen , M. Zhang , A. Duan , W. Qin , K. Zen , Z. Liu , Diabetes 2018, 67, 717. 29242313 10.2337/db17-0755

[39] a) S. I. Landau , X. Guo , H. Velazquez , R. Torres , E. Olson , R. Garcia‐Milian , G. W. Moeckel , G. V. Desir , R. Safirstein , Kidney Int. 2019, 95, 797; 30904067 10.1016/j.kint.2018.11.042 PMC6543531

[40] R. Graf , X. Li , V. T. Chu , K. Rajewsky , Cell Rep. 2019, 26, 1098. 30699341 10.1016/j.celrep.2019.01.024 PMC6352712

[41] J. Xiong , L. Ran , Y. Zhu , Y. Wang , S. Wang , Y. Wang , Q. Lan , W. Han , Y. Liu , Y. Huang , T. He , Y. Li , L. Liu , J. Zhao , K. Yang , Theranostics 2022, 12, 5069. 35836796 10.7150/thno.72291 PMC9274747

[42] Y. Huang , J. Zhou , S. Wang , J. Xiong , Y. Chen , Y. Liu , T. Xiao , Y. Li , T. He , Y. Li , X. Bi , K. Yang , W. Han , Y. Qiao , Y. Yu , J. Zhao , Theranostics 2020, 10, 7384. 32641998 10.7150/thno.45455 PMC7330852

[43] Y. Liu , X. Bi , J. Xiong , W. Han , T. Xiao , X. Xu , K. Yang , C. Liu , W. Jiang , T. He , Y. Yu , Y. Li , J. Zhang , B. Zhang , J. Zhao , Mol. Ther. 2019, 27, 1051. 30853453 10.1016/j.ymthe.2019.02.009 PMC6520492

[44] Y. Fan , W. Xiao , K. Lee , F. Salem , J. Wen , L. He , J. Zhang , Y. Fei , D. Cheng , H. Bao , Y. Liu , F. Lin , G. Jiang , Z. Guo , N. Wang , J. C. He , J. Am. Soc. Nephrol. 2017, 28, 2007. 28137829 10.1681/ASN.2016091001 PMC5491287

[45] J. N. Wang , F. Wang , J. Ke , Z. Li , C. H. Xu , Q. Yang , X. Chen , X. Y. He , Y. He , X. G. Suo , C. Li , J. T. Yu , L. Jiang , W. J. Ni , J. Jin , M. M. Liu , W. Shao , C. Yang , Q. Gong , H. Y. Chen , J. Li , Y. G. Wu , X. M. Meng , Sci. Transl. Med. 2022, 14, abk2709. 10.1126/scitranslmed.abk2709 35417191

[46] L. Markó , E. Vigolo , C. Hinze , J. K. Park , G. Roël , A. Balogh , M. Choi , A. Wübken , J. Cording , I. E. Blasig , F. C. Luft , C. Scheidereit , K. M. Schmidt‐Ott , R. Schmidt‐Ullrich , D. N. Müller , J. Am. Soc. Nephrol. 2016, 27, 2658. 26823548 10.1681/ASN.2015070748 PMC5004652

[47] M. E. Ritchie , B. Phipson , D. Wu , Y. Hu , C. W. Law , W. Shi , G. K. Smyth , Nucleic Acids Res. 2015, 43, 47. 10.1093/nar/gkv007 PMC4402510 25605792

[48] Y. Zhou , B. Zhou , L. Pache , M. Chang , A. H. Khodabakhshi , O. Tanaseichuk , C. Benner , S. K. Chanda , Nat. Commun. 2019, 10, 1523. 30944313 10.1038/s41467-019-09234-6 PMC6447622

[49] Y. Wang , Y. Li , Z. Chen , Y. Yuan , Q. Su , K. Ye , C. Chen , G. Li , Y. Song , H. Chen , Y. Xu , Cell Death Dis. 2022, 13, 693. 35941120 10.1038/s41419-022-05138-4 PMC9360039

[50] I. Korsunsky , N. Millard , J. Fan , K. Slowikowski , F. Zhang , K. Wei , Y. Baglaenko , M. Brenner , P. R. Loh , S. Raychaudhuri , Nat. Methods 2019, 16, 1289. 31740819 10.1038/s41592-019-0619-0 PMC6884693

