# Supplementary material for: SREBP1c‐Mediated Transcriptional Repression of YME1L1 Contributes to Acute Kidney Injury by Inducing Mitochondrial Dysfunction in Tubular Epithelial Cells
Source: Adv Sci (Weinh). 2024 Dec 16;12(6):2412233. doi: 10.1002/advs.202412233 (PMC11809329; doi:10.1002/advs.202412233)
Supplement: Supplementary file 1 — Supporting Information [file ADVS-12-2412233-s001.docx]

Supporting Information

SREBP1c-mediated Transcriptional Repression of YME1L1 Contributes to Acute Kidney Injury by Inducing Mitochondrial Dysfunction in Tubular Epithelial Cells

*Wang Xin, Jie Zhou, Yuzhu Peng, Shuiqin Gong, Wenhao Liao, Yaqin Wang, Xixin Huang, Yang Mao, Mengying Yao, Shaozong Qin, Jiachuan Xiong, Yan Li, Qigang Lan, Yinghui Huang*, Jinghong Zhao**

**
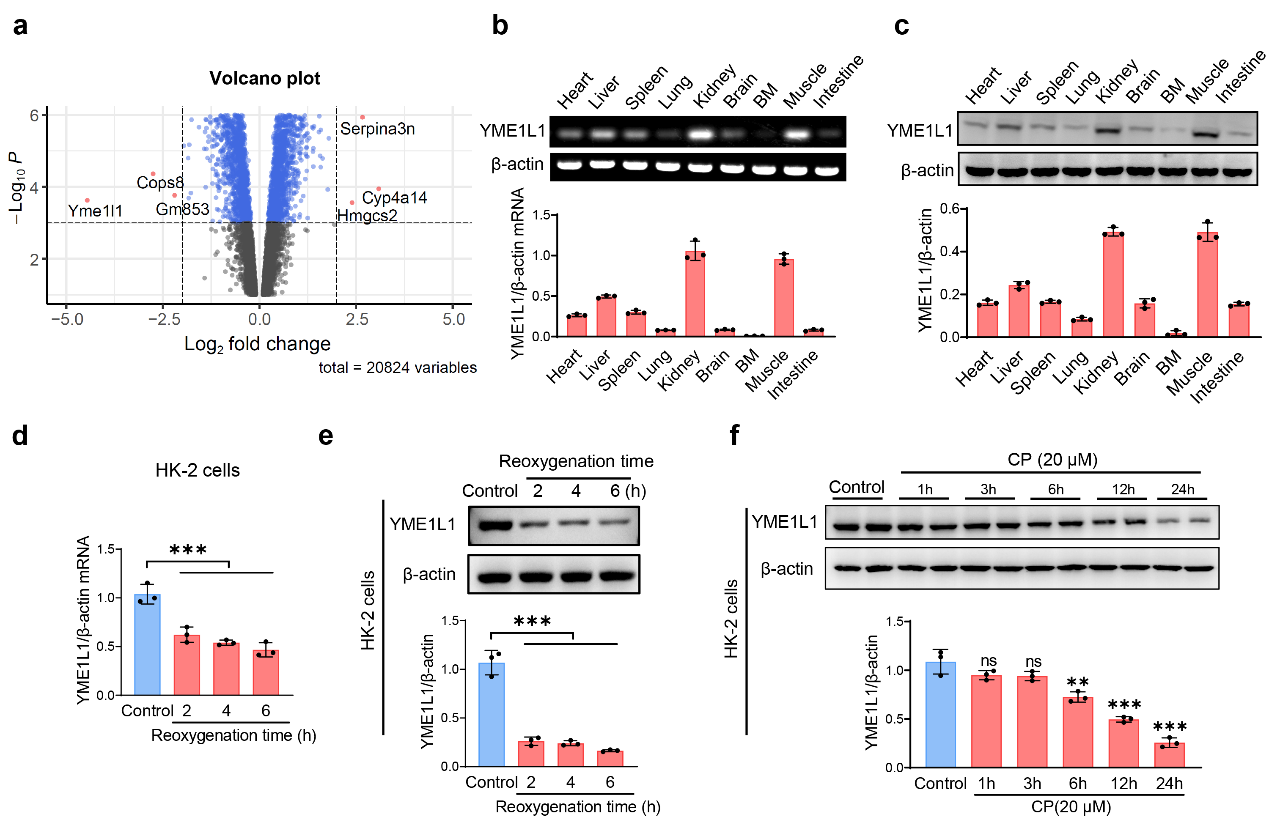
Figure S1. YME1l1 expression is downregulated in AKI.** (**a**) Volcano plot of RNA sequencing showing differentially expressed genes in the kidney tissues of control and AKI mice. (**b** and **c**) qPCR and Western blot analysis of YME1L1 expression in the selected mouse tissues, including heart, liver, spleen, lung, kidney, brain, bone marrow (BM), muscle and intestine (n = 3). (**d** and **e**) qPCR and Western blot analysis of YME1L1 expression in HK-2 cells treated with hypoxia-reoxygenation injury for the indicated time (n = 3). (**f**) Western blot analysis of YME1L1 expression in HK-2 cells treated with cisplatin (CP, 20μM) for the indicated time (n = 3). Data are shown as means ± SD and were analyzed one-way ANOVA (**d-f**). ns: no significance. ** *P* < 0.01, *** *P* < 0.001.

**
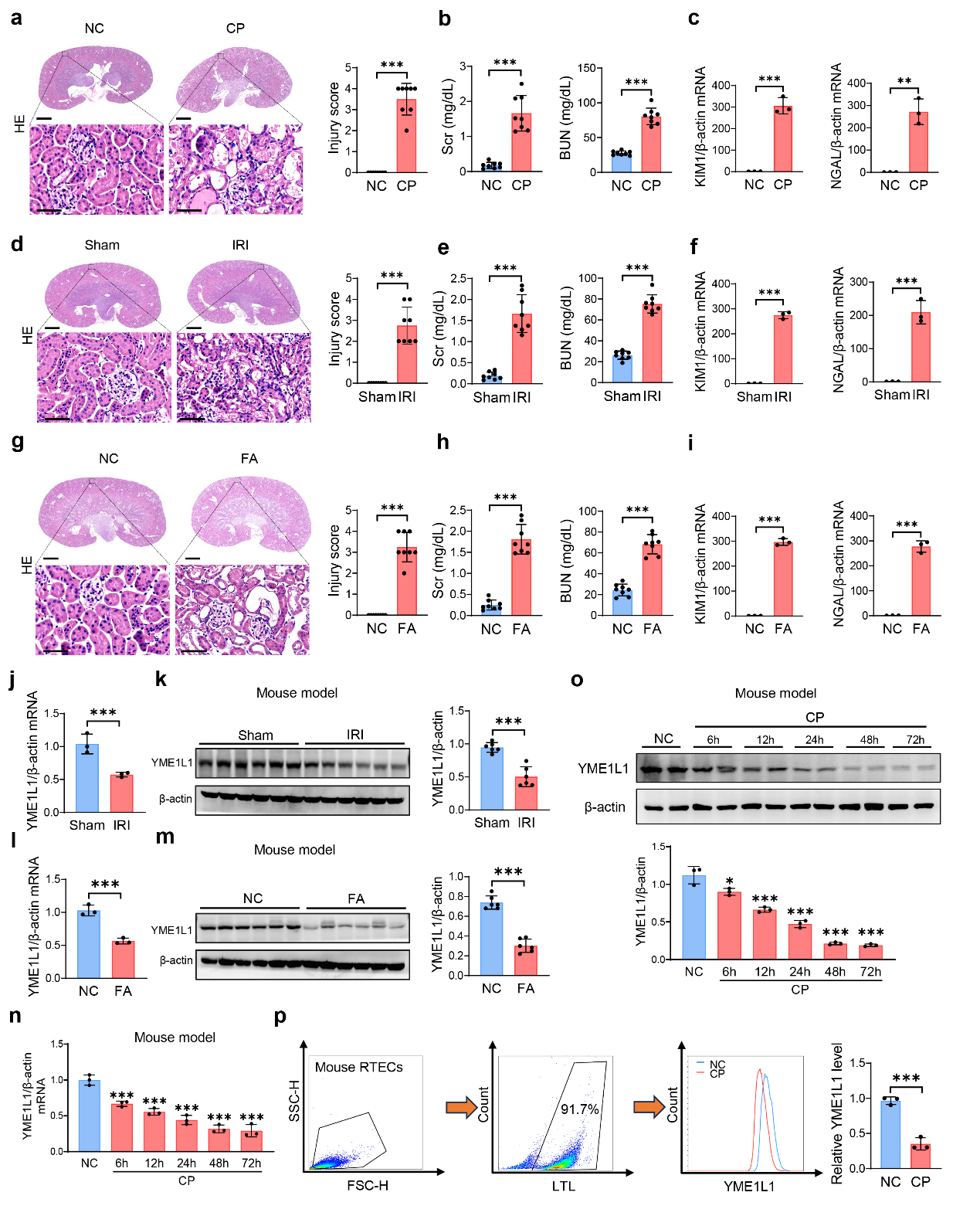
Figure S2. YME1l1 expression is downregulated in mouse kidney tissue and primary renal tubular epithelial cells under AKI conditions.** (**a**-**m**) Mouse models of AKI were induced by CP (25mg/kg), folic acid (FA, 250mg/kg) and ischemia-reperfusion injury (IRI), n = 8 mice in each group. Mice were sacrificed for HE staining in kidney tissues (**a**, **d**, **g,** Scale bar, 1 mm, (top) and 50 μm (bottom)), detection of the levels of Scr and BUN (**b**, **e**, **h**) (n = 8), detection of KIM1 and NGAL expression (**c**, **f**, **i**) (n = 3), qPCR and Western blot analysis of YME1L1(**j**-**m**). (**n** and **o**) Cisplatin-treated mice were sacrificed at indicated time. The kidney tissues were collected for Western blot and qPCR analysis of YME1L1 expression (n = 3). (**p**)Primary renal tubular epithelial cells were isolated from cisplatin-treated mice and were used to assess YME1L1 expression by Flow cytometry. Lotus lectin (LTL) was used to label proximal renal tubular epithelial cells (n = 3). Data are shown as means ± SD and were analyzed by two-tailed unpaired Student's *t*-test (**a**-**m** and **p**) or one-way ANOVA (**n** and **o**). * *P* < 0.05, ** *P* < 0.01, *** *P* < 0.001.

**
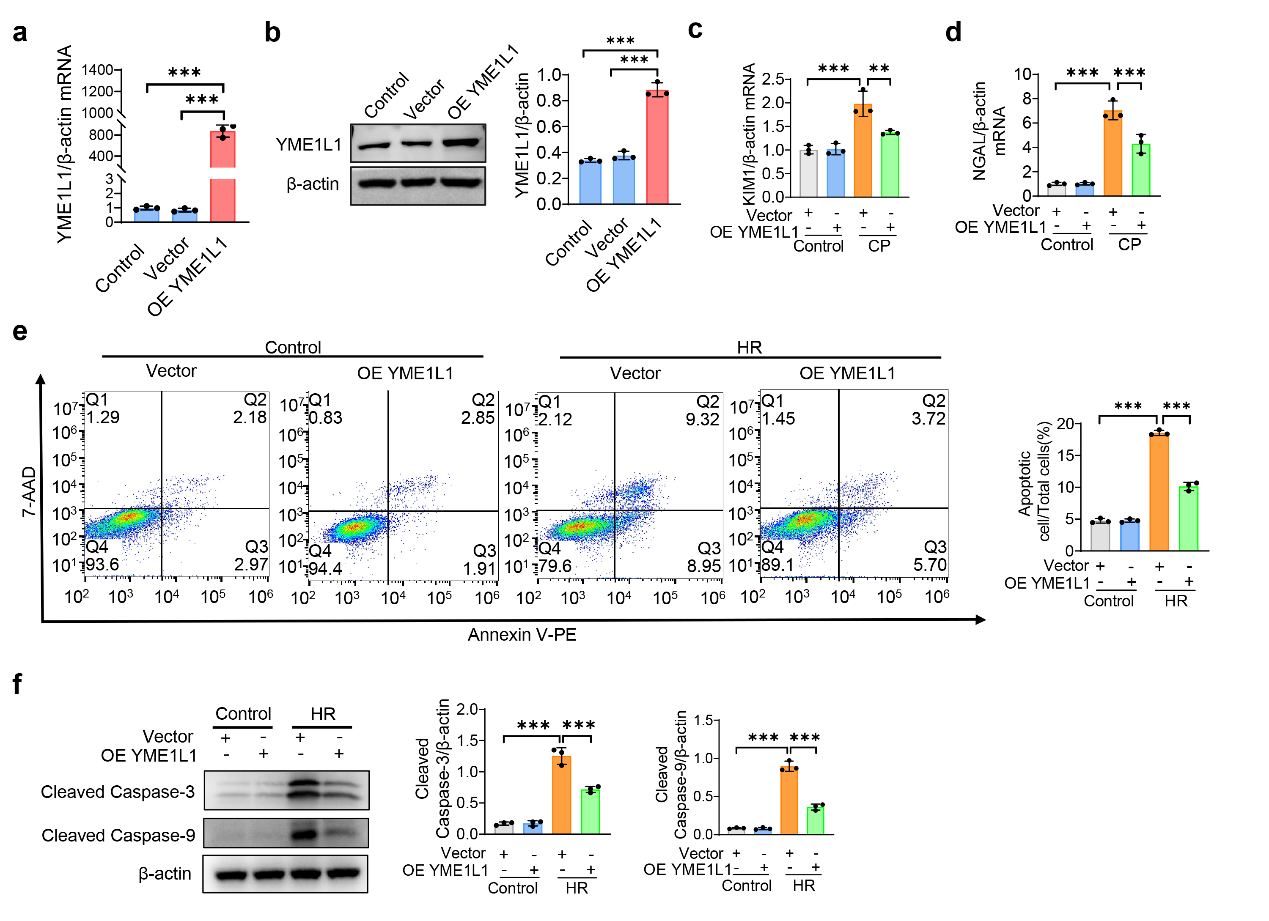
Figure S3. YME1L1 attenuates cisplatin-induced HK-2 cells injury *in vitro*.** (**a**-**b**) HK-2 cells were transfected with control or Vector or YME1L1 overexpression plasmids, then qPCR (**a**) and Western blot (**b**) were conducted to analyze the expression of YME1L1 (n = 3). (**c** and **d**) HK-2 cells were transfected with Vector or YME1L1 overexpression plasmids and then treated with control or 20 μM CP for 24 hours, and then cells were harvested for qPCR analysis of KIM1 and NGAL expressions (n = 3). (**e** and **f**) HK-2 cells were transfected with Vector or YME1L1 overexpression plasmids and then treated with hypoxia for 24 hours and reoxygenation 6 hours. Cells were harvested for flow cytometry analysis of apoptotic cells (**e**) (n = 3) and Western blot analysis of Cleaved caspase-3 and Cleaved caspase-9 (**f**) (n = 3). Data are shown as means ± SD and were analyzed by one-way ANOVA (**a**-**f**). ** *P* < 0.01, *** *P* < 0.001. flow cytometry analysis of apoptotic cells

**
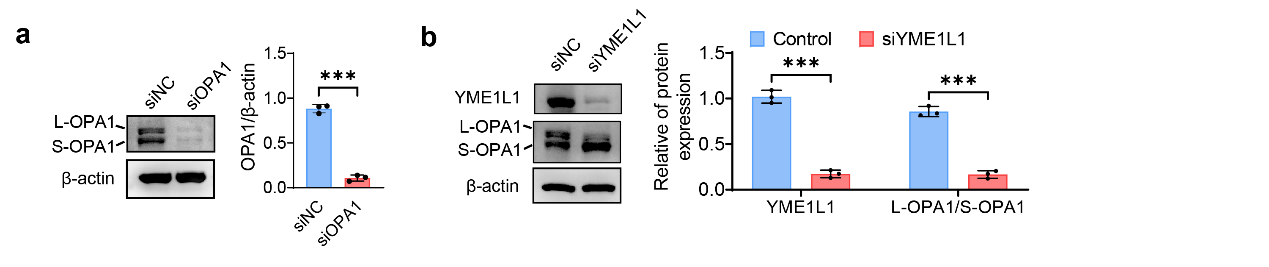
Figure S4. YME1L1 ameliorates cisplatin-induced mitochondrial dysfunction via restoring OPA1-mediated mitochondrial energy metabolism.** (**a**) HK-2 cells were transfected with siNC or siOPA1 RNA for detection of OPA1 expression (n = 3). (**b**) HK-2 cells were transfected with siNC or siYME1L1, and then cells were harvested for Western blot analysis of YME1L1 and L-OPA1/S-OPA1 expression (n = 3). Data are shown as means ± SD and were analyzed by two-tailed unpaired Student's *t*-test (**a** and **b**). *** *P* < 0.001.

**
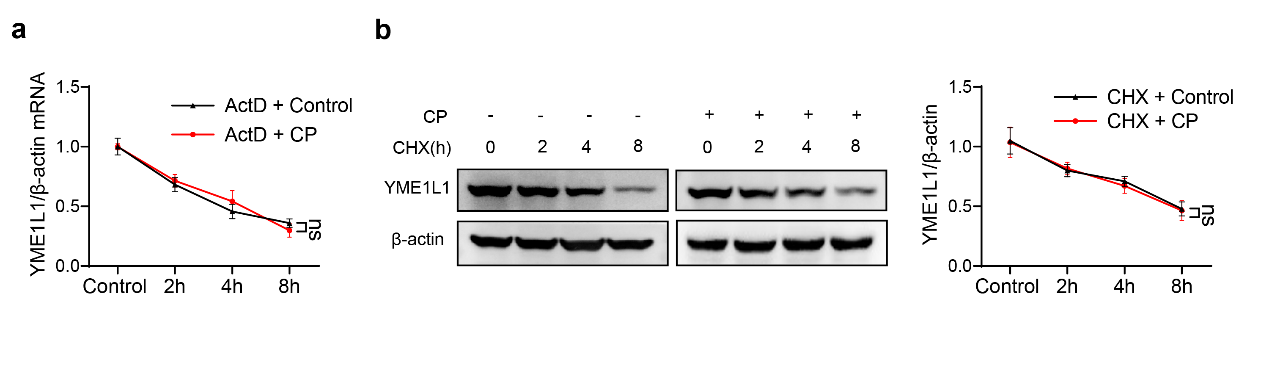
Figure S5.** **Cisplatin can regulate neither the mRNA stability nor the protein degradation of YME1L1.** (**a**) HK-2 cells were treated with a transcriptional inhibitor actinomycin D (ActD, 0.5 μg/ml) for various times in the absence or presence of cisplatin. YME1L1 mRNA expression was determined using qPCR (n = 3). (**b**) HK-2 Cells were treated with a translational inhibitor cycloheximide (CHX, 50 μg/ml) time-dependently in the absence or presence of cisplatin. YME1L1 protein expression was detected using Western blot (n = 3). Data are shown as means ± SD and were analyzed by two-tailed unpaired Student's *t*-test (**a** and **b**). ns: no significance.

**
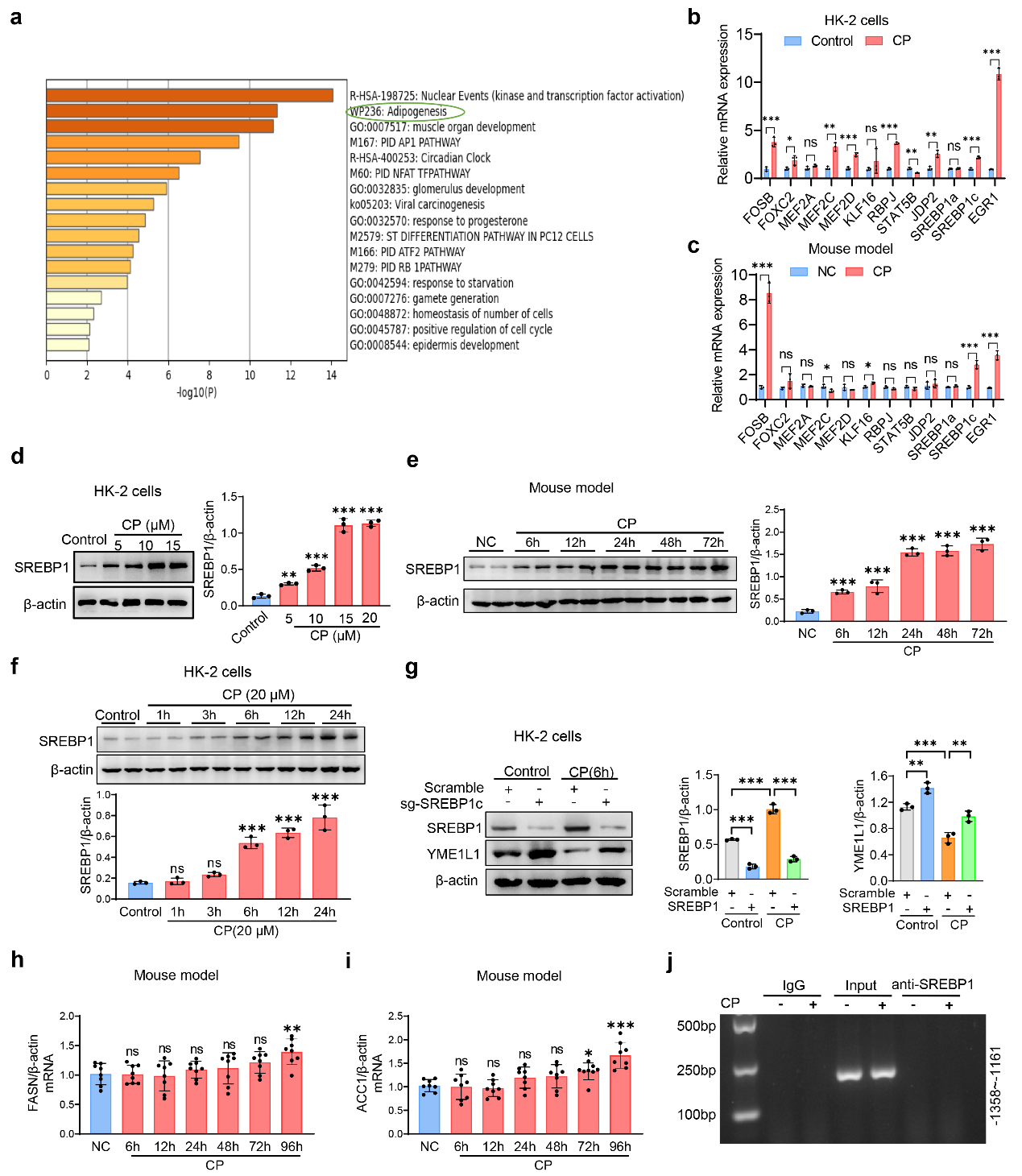
Figure S6.** **Upstream transcription factors of YME1L1 were predicted and screened.** (**a**) GO analysis of the biological processes involved in the predicted transcription factors by Metascape. (**b** and **c**) qPCR screening of the adipogenesis-associated transcription factors in HK-2 cells (**b**) and kidney tissues (**c**) (n = 3). (**d**) SREBP1 expression was analyzed by Western blot in HK-2 cells treated with different concentrations of CP for 24 hours (n = 3). (**e**) Western blot analysis of SREBP1 expression in mouse models of AKI induced by CP (25 mg/kg) treated with indicated time (n = 3). (**f**) Western blot analysis of SREBP1 expression in HK-2 cells treated with CP (20 μM) for the indicated time (n = 3). (**g)** HK-2 cells were transfected with sgRNA (Scramble or SREBP1c) in combination with control or 20 μM CP for 6 hours, then YME1L1 and SREBP1 expression was analyzed by Western blot (n = 3). (**h** and **i**) Mice were treated with CP (25mg/kg) for indicated time, and the renal tissues were detected FASN and ACCC1 expression by qPCR (n = 8). (**j**) HK-2 cells were treated with control or CP for 24 hours, and cells were collected for ChIP assay. IgG was used as a negative control. The primers covering the YME1L1 primers covering this region (-1358 ~ -1161) were used as a negative control. Data are shown as means ± SD and were analyzed by two-tailed unpaired Student's *t*-test (**b** and **c**) or one-way ANOVA (**d-i**). ns: no significance. * *P* < 0.05, ** *P* < 0.01, *** *P* < 0.001.

**
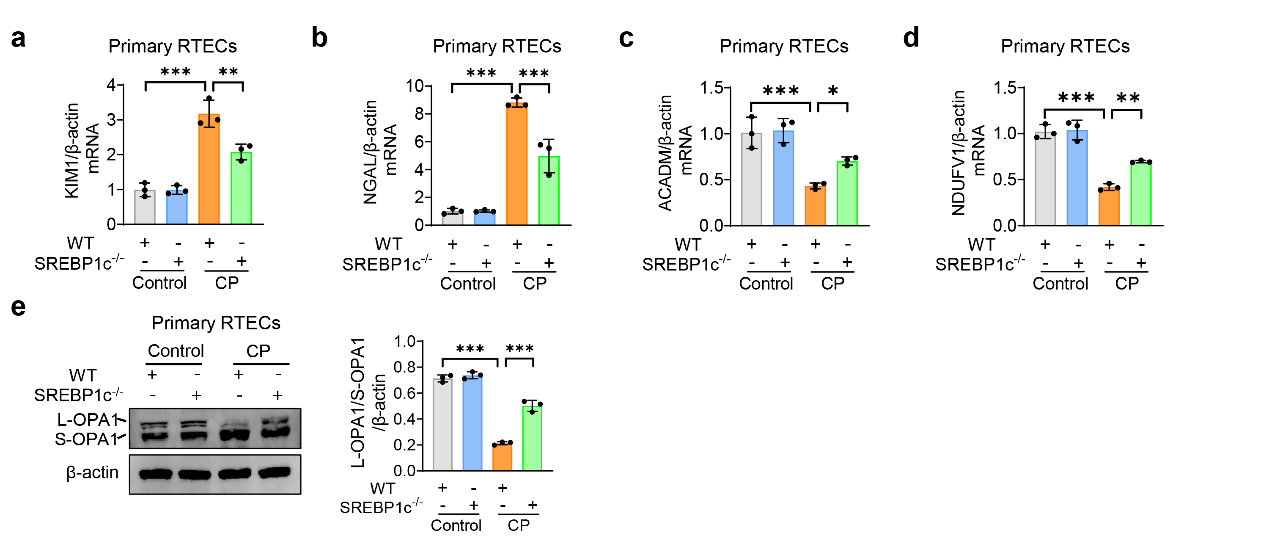
Figure S7.** **Knockout of SREBP1c alleviates cisplatin-induced mitochondrial dysfunction and apoptosis *in vitro*.** (**a**-**e**) Primary RTECs from WT and SREBP1c knockout (KO) mice were treated with control or 20 μM CP for 24 hours. Cells were harvested for qPCR analysis of KIM1 (**a**), NGAL (**b**), ACADM (**c**) and NDUFV1 (**d**) expression, and Western blot analysis of OPA1 (**e**) (n = 3). Data are shown as means ± SD and were analyzed by one-way ANOVA (**a**-**e**). * *P* < 0.05, ** *P* < 0.01, *** *P* < 0.001.

**
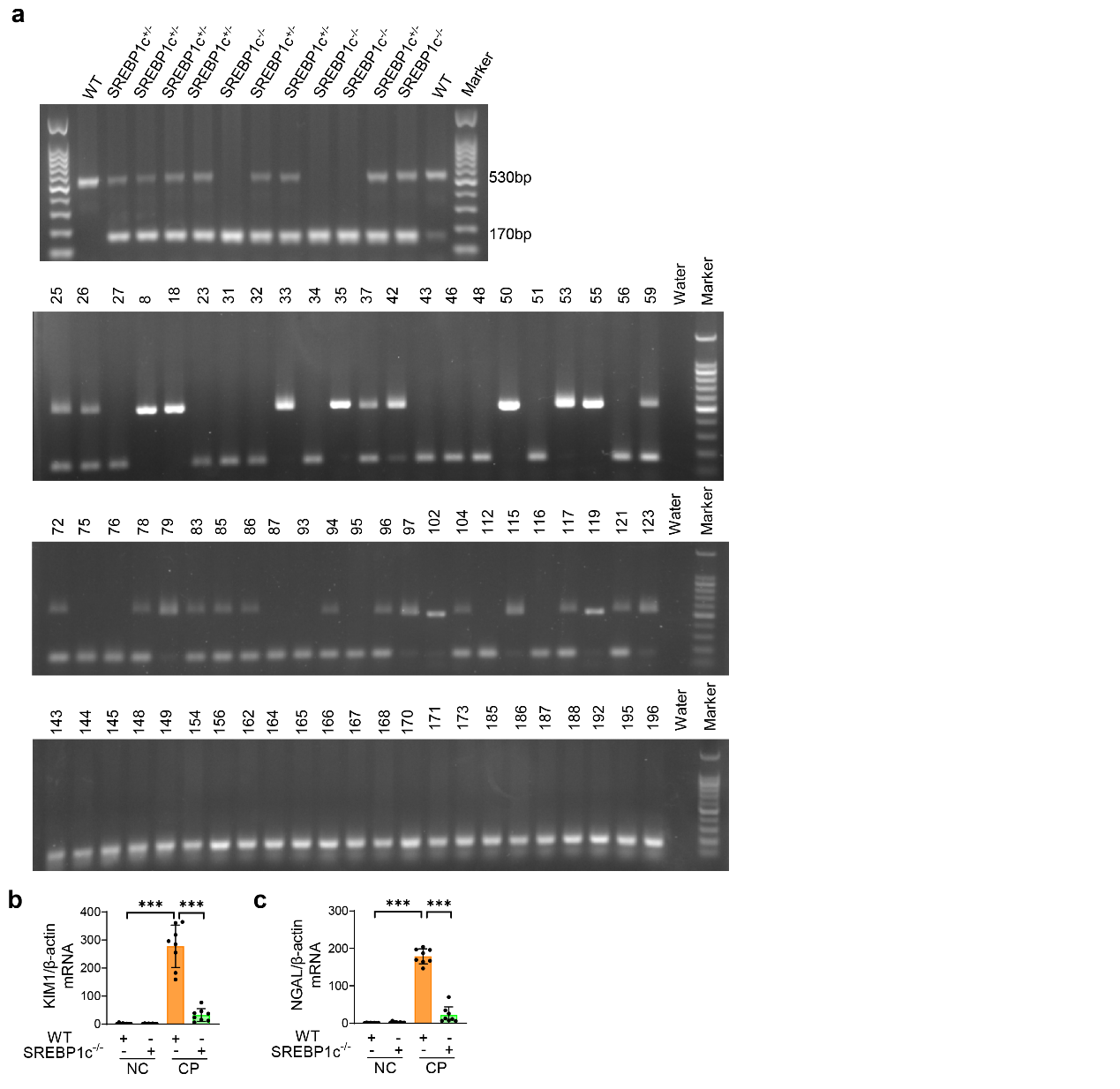
Figure S8.** **Knockout of SREBP1c improves cisplatin-induced AKI.** (**a**) Validation of WT and SREBP1c KO by PCR using mouse tail DNA and specific primers. 530 bp bands represent the WT allele, 170 bp bands represent the SREBP1c KO Homozygous gene, and 170 and 530 bp bands represent the SREBP1c KO heterozygous alleles. (**b** and **c**) WT and SREBP1c^-/-^ mice were treated with a single dose of control or CP (25 mg/kg) and then sacrificed after 3 days for qPCR analysis of KIM1 and NGAL expression. n = 8 mice in each group. Data are shown as mean ± SD and were analyzed by one-way ANOVA (**b** and **c**). *** *P* < 0.001.

**
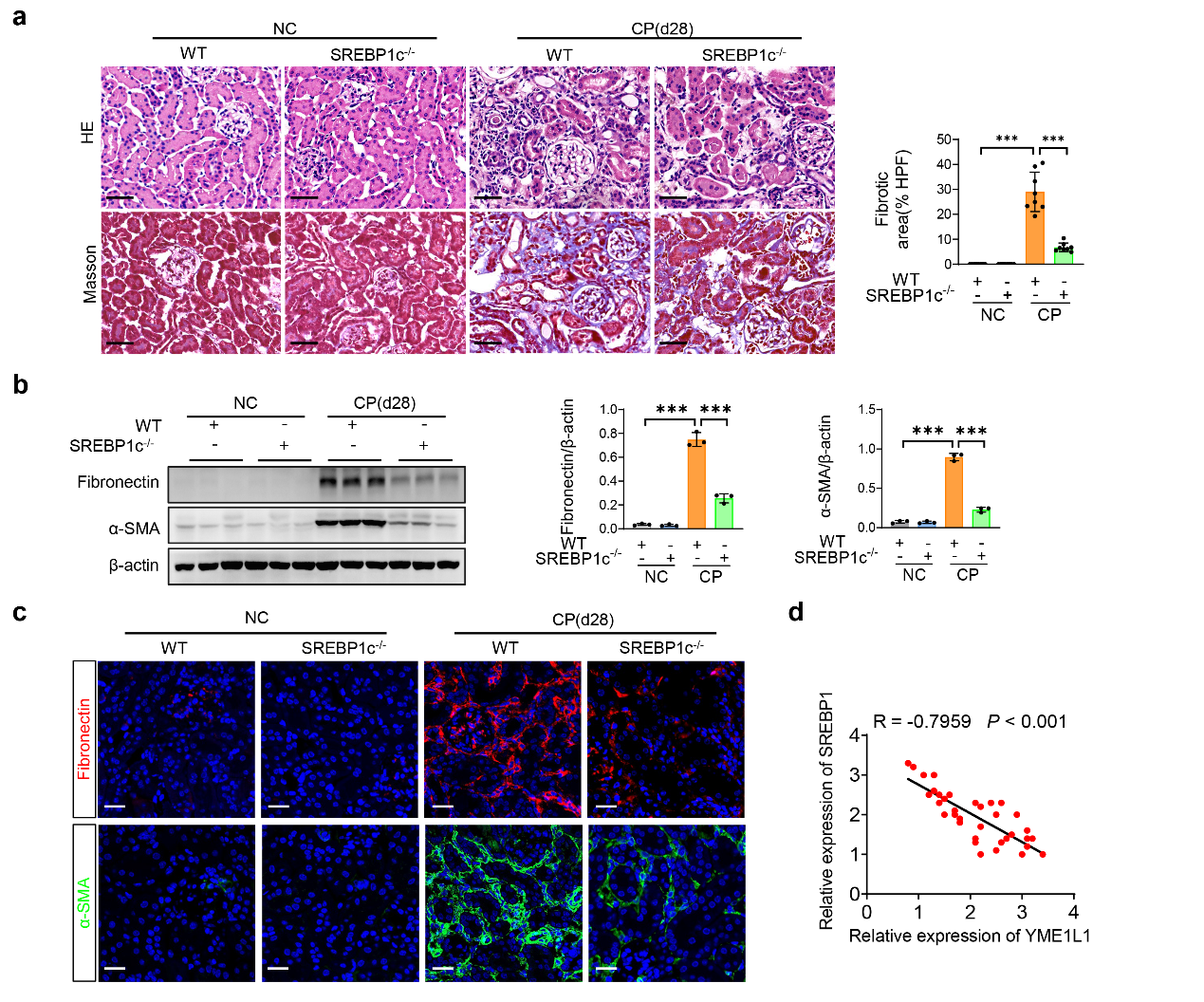
Figure S9.** **Knockout of SREBP1c prevents cisplatin-induced chronic renal fibrosis *in vivo*.** (**a**-**c**) AKI to CKD mouse model was induced by 15 mg/kg of cisplatin after 28 days, then kidney tissues were collected for HE and Masson staining (**a**), Western blot analysis of Fibronectin and α-SMA expression (**b**) (n = 3) and immunofluorescence staining of Fibronectin and α-SMA (**c**, Scale bar, 20 μm). (**d**) Correlation analysis between YME1L1 and SREBP1 staining intensity. Data are shown as mean ± SD and were analyzed by one-way ANOVA (**a** and **b**) and Spearman’s rank correlation test (**d**). *** *P* < 0.001.

**
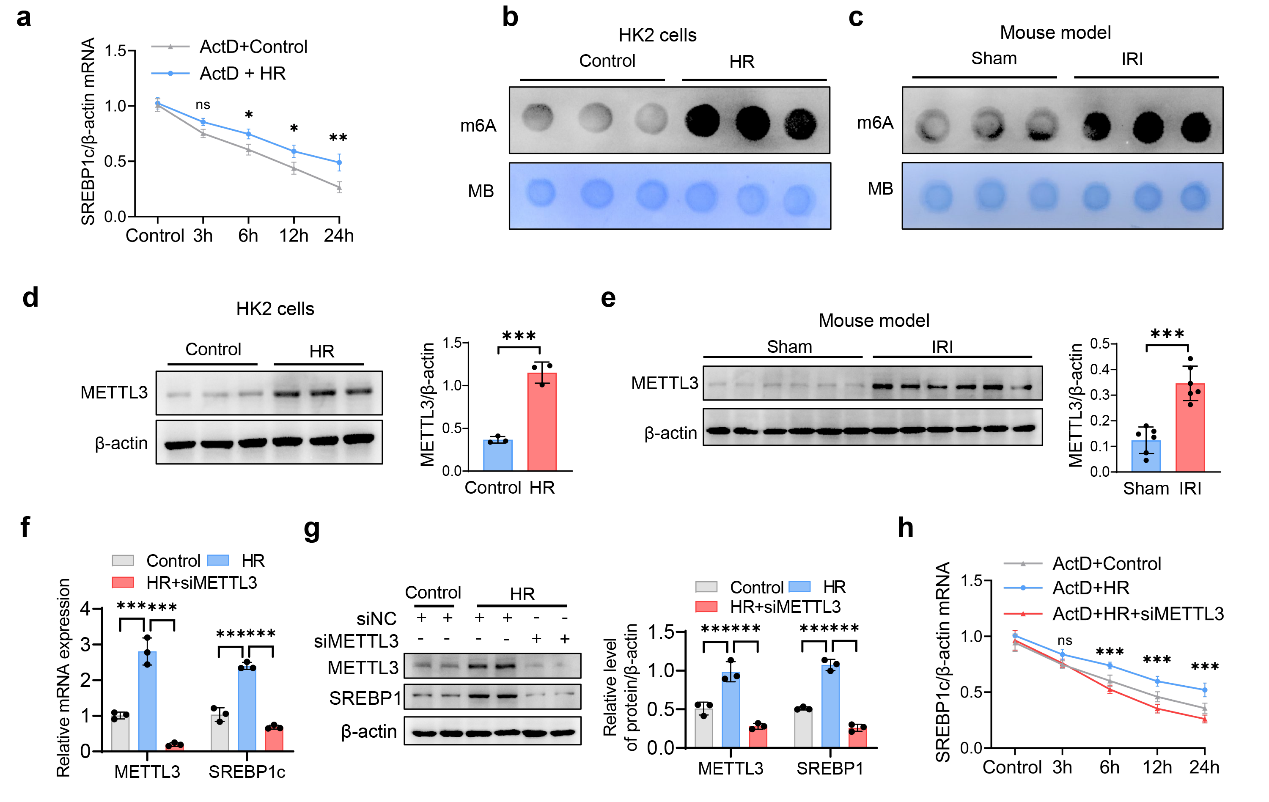
**

**Figure S10. METTL3 regulates m6A enrichment and stability of SREBP1c mRNA.** (**a**) HK-2 cells received treatment with the transcriptional inhibitor Actd (0.5 μg/ml) of different durations under normoxia or HR conditions. SREBP1c mRNA expression was determined using qPCR (n = 3). (**b** and **c**) m6A mRNA methylation were assessed via m6A dot blot in HK-2 cells under normoxia or HR conditions. (n = 3) and mouse model of AKI induced by IRI. n = 8 mice in each group. (**d** and **e**) The expression of METTL3 was analyzed by Western blot in HK-2 cells under normoxia or HR conditions (n = 3) and mouse model of AKI induced by IRI. n = 8 mice in each group. (**f** and **g**) HK-2 cells were transfected with siMETTL3 or siNC and then exposed to HR injury for qPCR and Western blot analysis of METTL3 and SREBP1 expression (n = 3). (**h**) HK-2 cells were transfected with siMETTL3 or siNC and treated with Actd for indicated time under normoxia or HR conditions. Cells were collected for qPCR analysis of SREBP1c expression. ns: no significance, * *P* < 0.05, *** *P* < 0.001 versus Actd-siMETTL3 group with CP treatment (n = 3). Data are shown as means ± SD and were analyzed by two-way ANOVA (**a** and **h**), two-tailed unpaired Student's *t*-test (**d** and **e**) or one-way ANOVA (**f** and **g**). ns: no significance. * *P* < 0.05, ** *P* < 0.01, *** *P* < 0.001.

**
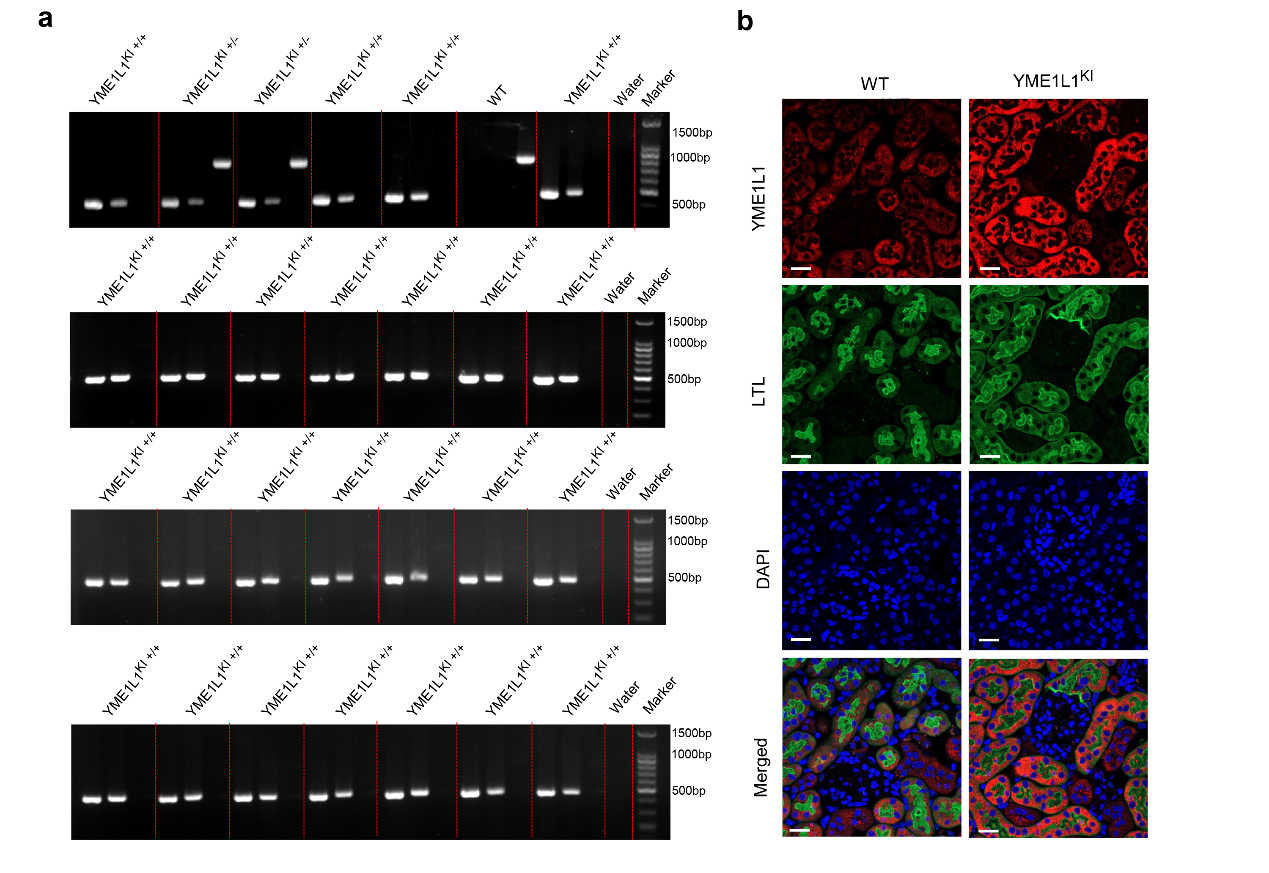
Figure S11.** **Identification of renal tubule specific knock-in YME1L1 mice.** Validation of WT and renal tubule-specific knock-in YME1L1 mice (YME1L1^KI^) by PCR using mouse tail DNA and specific primers. 825 bp bands represent the WT allele, 467 and 475 bp bands represent the YME1L1^KI^ Homozygous alleles, and 467, 475 and 825 bp bands represent the YME1L1^KI^ heterozygous alleles. (**b**) Representative immunofluorescence staining of YME1L1 (red) expression in the kidney of WT or YME1L1^KI^ mice. Lotus lectin (LTL, green) was used to label proximal tubules. Scale bar, 20 μm.

**
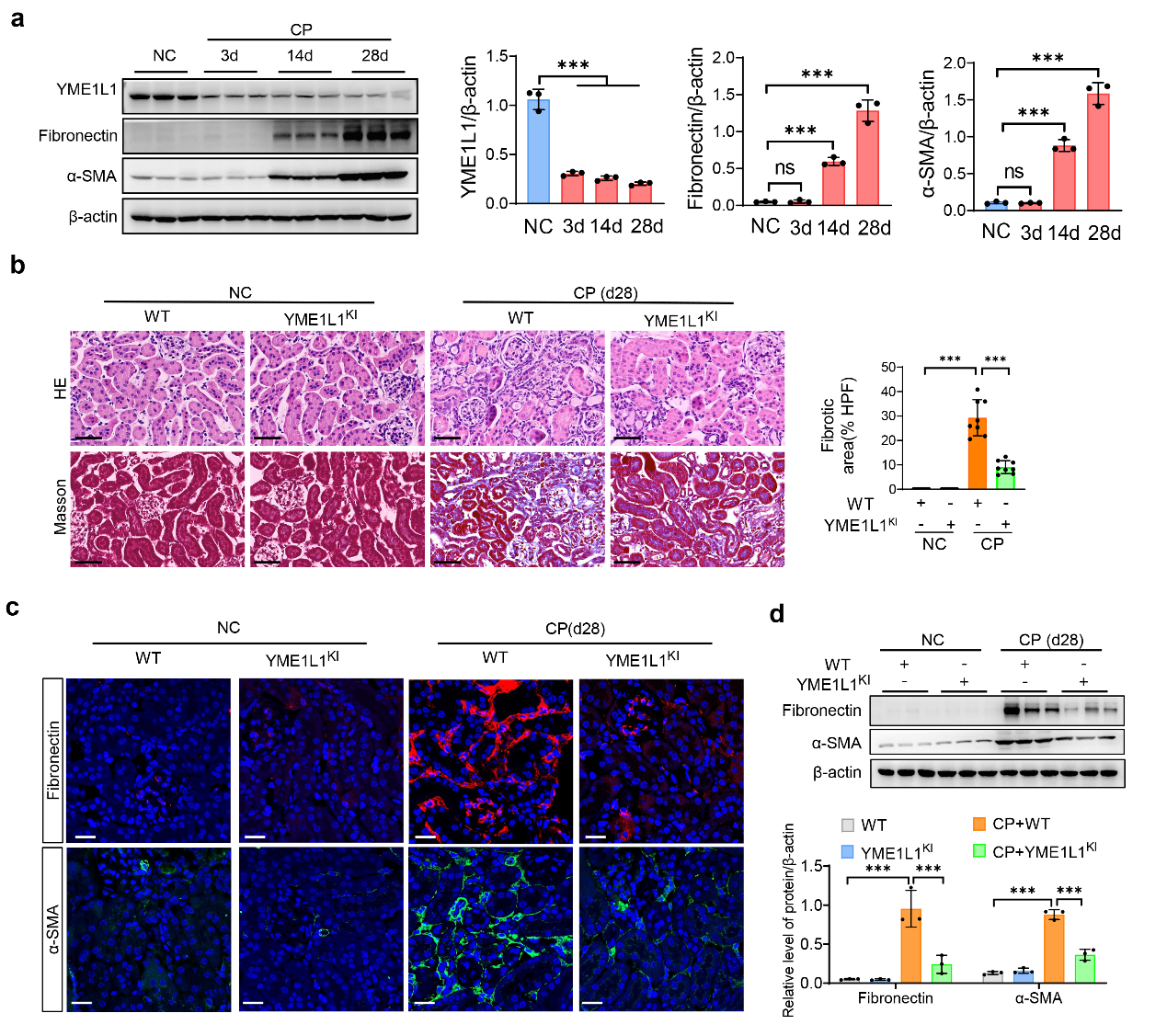
Figure S12.** **YME1L1 overexpression attenuates cisplatin-induced chronic renal fibrosis.** (**a**) Mice were injected with 15 mg/kg of cisplatin and sacrificed at different time points, then kidney tissues were collected for Western blot of YME1L1, Fibronectin and α-SMA expression (n = 3). (**b**-**d**) AKI to CKD mouse model was induced in WT and YME1L1^KI^ mice by 15 mg/kg of cisplatin after 28 days, then kidney tissues were collected for HE and Masson staining (**b**, Scale bar, 50 μm), immunofluorescence staining and Western blot analysis of Fibronectin and α-SMA (**c** and **d**, Scale bar, 20 μm). n = 8 mice in each group. Data are shown as mean ± SD and were analyzed by one-way ANOVA (**a, b** and **d**). ns: no significance. *** *P* < 0.001.

**
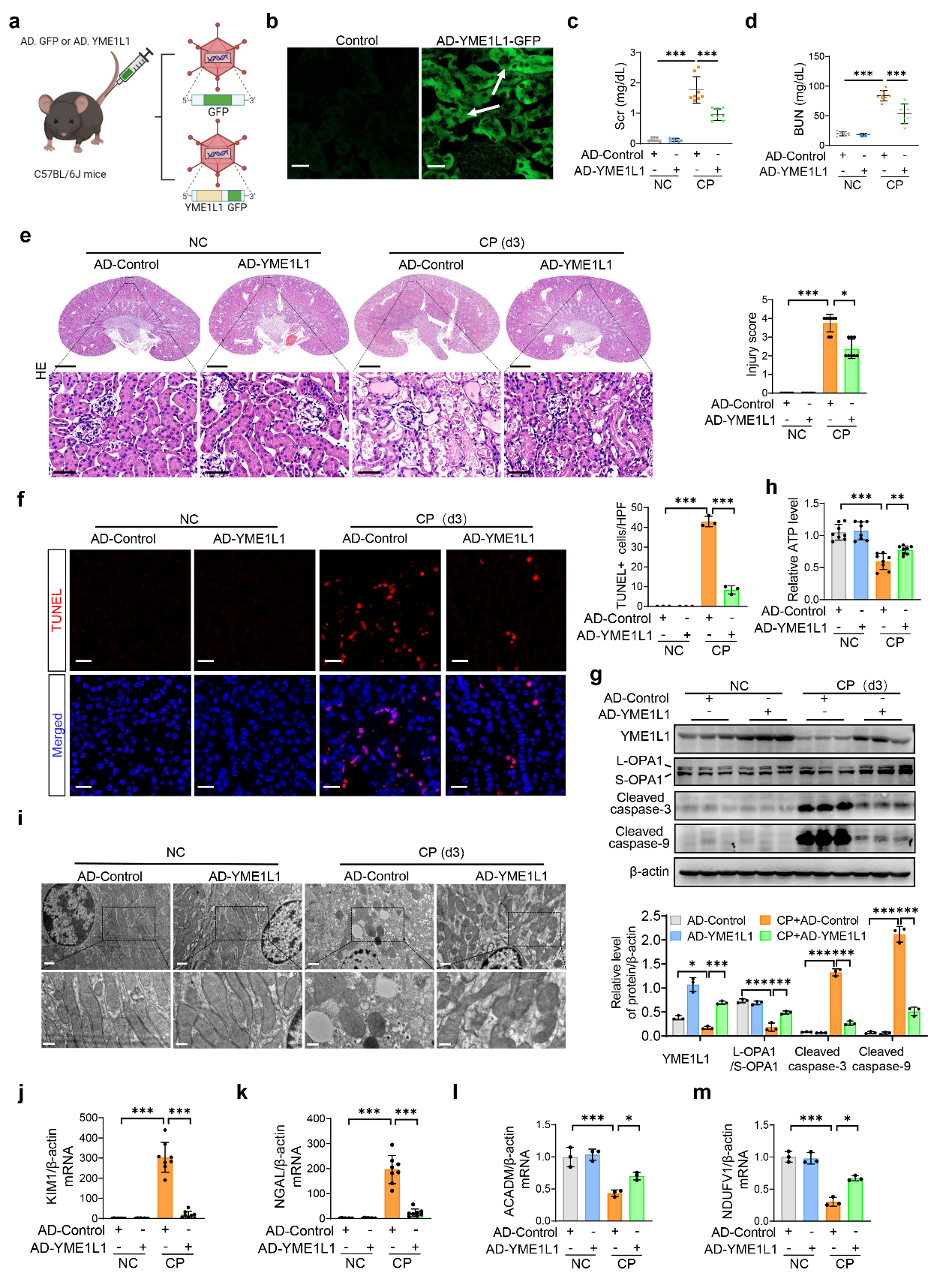
Figure S13. Exogenous YME1L1 relieves cisplatin-induced AKI.** (**a**) Schematic diagram of C57BL/6J mice injected with recombinant adenovirus expressing YME1L1 (Ad-YME1L1). (**b**) Representative fluorescence images of kidney tissues from Control and AD-YME1L1. Bright Green fluorescent protein (GFP) fluorescence of mouse kidney tissue indicated expression of YME1L1, Scale bar, 20 μm. (**c**-**m**) Mice were injected intravenously with Ad-Control or AD-YME1L1, then injected intraperitoneally with a single dose of saline or 25 mg/kg cisplatin. They were sacrificed on day 3 and serum was collected to detect the levels of Scr and BUN (**c** and **d**).The kidney tissues were collected to assess HE staining and scoring of renal tubular injury (**e**, scale bar, 1 mm (top) and 50 μm (bottom)), TUNEL staining (**f**, scale bar, 20 μm), Western blot analysis of the expressions of YME1L1, L-OPA1/S-OPA1, Cleaved caspase-3 and Cleaved caspase-9 (**g**), ATP levels (**h**) (n = 8), TEM observation (**i**, scale bar, 1 μm (top) and 0.5 μm (bottom)), and qPCR analysis of KIM1, NGAL, ACADM and NDUFV1 expression (**j**-**m**). Data are shown as means ± SD and were analyzed by one-way ANOVA (**c**-**h** and **j-m**). * *P* < 0.05, ** *P* < 0.01, *** *P* < 0.001.

**
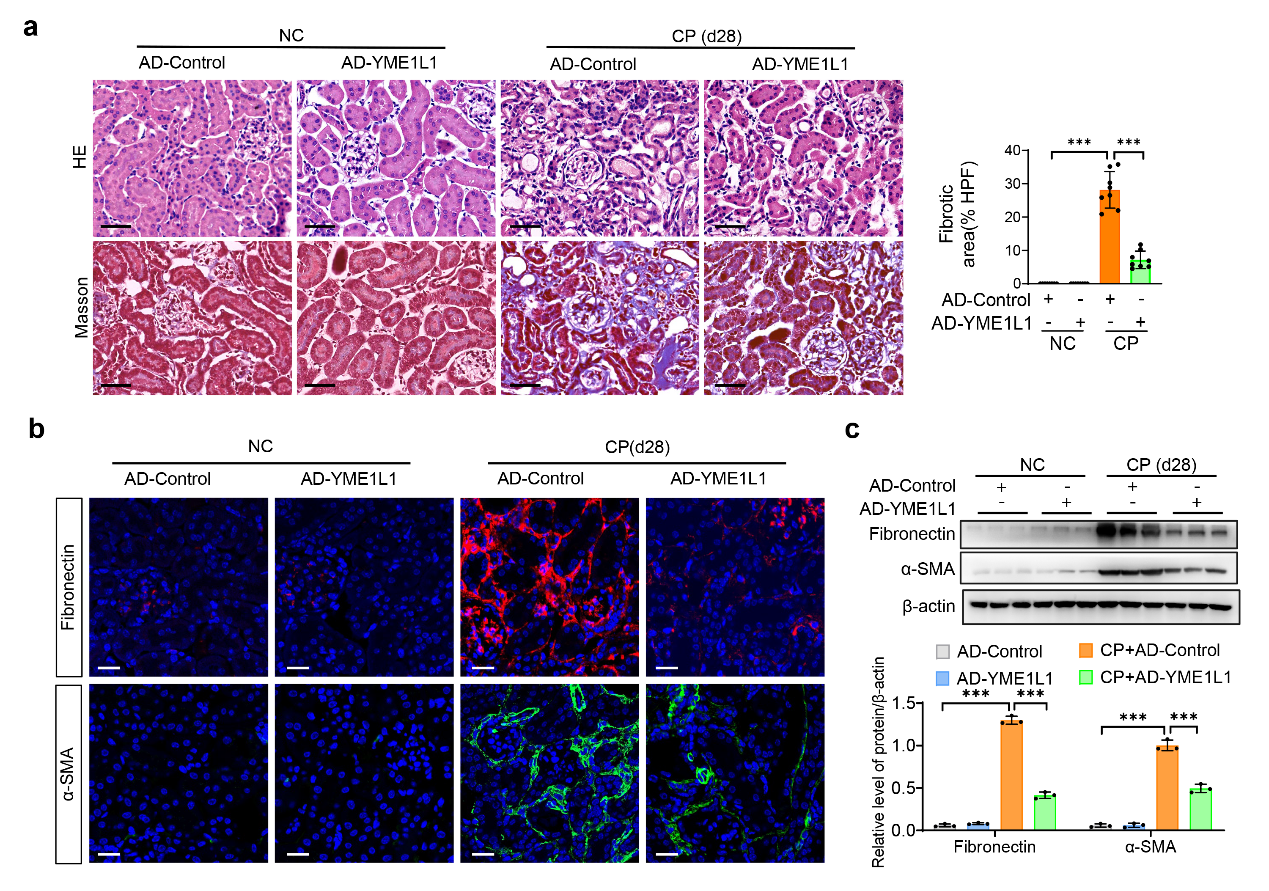
Figure S14.** **Exogenous YME1L1 attenuates cisplatin-induced chronic renal fibrosis.** (**a**-**c**) AKI to CKD mouse model was induced in AD-control and AD-YME1L1 mice by 15 mg/kg of cisplatin after 28 days, then kidney tissues were collected for HE and Masson staining (**a**, Scale bar, 50 μm), immunofluorescence staining and Western blot analysis of Fibronectin and α-SMA expression (**b** and **c**, Scale bar, 20 μm) (**c**). n = 8 mice in each group. Data are shown as mean ± SD and were analyzed by one-way ANOVA (**a** and **c**). *** *P* < 0.001.

**Table S1.** The basic characteristic of the included AKI patients

| Patients No. | Age (years) | Sex | Pathologic diagnosis | eGFR (ml/min per 1.73 m^2^) | Serum creatine (mg/dL) |
| --- | --- | --- | --- | --- | --- |
| 1 | 26 | Male | Acute tubular necrosis | 73 | 1.329 |
| 2 | 28 | Male | Acute tubular necrosis | 81 | 1.208 |
| 3 | 75 | Male | Acute tubular necrosis | 56 | 1.251 |
| 4 | 21 | Male | Acute tubular necrosis | 12 | 5.940 |
| 5 | 33 | Female | Acute tubular necrosis | 46 | 1.491 |
| 6 | 61 | Female | Acute tubular necrosis | 15 | 3.122 |
| 7 | 24 | Male | Acute tubular necrosis | 19 | 4.074 |
| 8 | 21 | Male | Acute tubular necrosis | 10 | 7.020 |
| 9 | 48 | Female | Acute tubular necrosis | 22 | 2.522 |
| 10 | 24 | Male | Acute tubular necrosis | 39 | 2.243 |
| 11 | 64 | Male | Acute tubular necrosis | 10 | 5.340 |
| 12 | 56 | Female | Acute tubular necrosis | 5 | 7.606 |
| 13 | 29 | Male | Acute tubular necrosis | 26 | 3.088 |
| 14 | 32 | Male | Acute tubular necrosis | 4 | 13.748 |
| 15 | 50 | Female | Acute tubular necrosis | 14 | 3.554 |
| 16 | 59 | Male | Acute tubular necrosis | 74 | 1.083 |
| 17 | 51 | Female | Acute tubular necrosis | 13 | 3.687 |
| 18 | 30 | Male | Acute tubular necrosis | 74 | 1.290 |
| 19 | 47 | Male | Acute tubular necrosis | 4 | 12.694 |
| 20 | 57 | Male | Acute tubular necrosis | 18 | 3.554 |
| 21 | 63 | Male | Acute tubular necrosis | 5 | 10.443 |
| 22 | 39 | Male | Control | 111 | 0.826 |
| 23 | 21 | Female | Control | 122 | 0.707 |
| 24 | 44 | Male | Control | 118 | 0.651 |
| 25 | 33 | Female | Control | 123 | 0.558 |
| 26 | 55 | Male | Control | 99 | 0.823 |
| 27 | 22 | Male | Control | 127 | 0.796 |
| 28 | 46 | Female | Control | 105 | 0.683 |
| 29 | 52 | Female | Control | 100 | 0.684 |
| 30 | 28 | Female | Control | 123 | 0.617 |
| 31 | 28 | Male | Control | 124 | 0.761 |
| 32 | 46 | Female | Control | 108 | 0.625 |
| 33 | 24 | Male | Control | 130 | 0.733 |
| 34 | 26 | Male | Control | 124 | 0.789 |
| 35 | 22 | Female | Control | 136 | 0.512 |
| 36 | 45 | Male | Control | 121 | 0.607 |

**Table S2**. Transcription factors in the promoter region of YME1L1 predicted by JASPAR

| Transcription factors | | | |
| --- | --- | --- | --- |
| ZBTB32 | ZNF263 | KLF15 | KLF5 |
| FOSL2:: JUND | CREM | ATF7 | ATF3 |
| FOSL1::JUND | Creb5 | CREB3L4 | JDP2 |
| FOSL1::JUN | JUNB | EGR3 | ZNF384 |
| FOSL2::JUNB | EGR1 | EGR3 | TFDP1 |
| FOSB::JUNB | RBPJ | MEF2A | MEF2C |
| MEF2D | Zfx | Foxj3 | FOXK1 |
| FOXK2 | FOXP2 | FOXC2 | FOXF2 |
| FOXD2 | Stat5b | ZNF384 | ZNF460 |
| KLF16 | ZNF460 | E2F6 | MEF2A |

**Table S3.** Putative binding sequences of SREBP1 in YME1L1 promoter region

| Name | Score | Relative score | Start | End | Strand | Predicted binding sites |
| --- | --- | --- | --- | --- | --- | --- |
| *SREBP1* | 11.0995 | 0.91261 | -1887 | -1878 | + | ctcacgccag |
| *SREBP1* | 9.63245 | 0.880396 | -1689 | -1680 | - | ttcacgccat |
| *SREBP1* | 9.36357 | 0.874492 | -898 | -889 | + | gtcaccccgc |
| *SREBP1* | 8.68279 | 0.859543 | -214 | -205 | + | atcgcgccac |
| *SREBP1* | 7.83434 | 0.840912 | -871 | -862 | + | gtcacctccc |
| *SREBP1* | 7.50167 | 0.833607 | -1374 | -1365 | + | gacacccgac |
| *SREBP1* | 7.33078 | 0.829854 | 180 | 189 | - | ctcacccgcc |
| *SREBP1* | 6.92644 | 0.820976 | -1508 | -1499 | + | atcacctcgg |

**Table S4.** The primer sets for human qPCR

| Gene (human) | **Primer Sequence (5'-3')** | Product length |
| --- | --- | --- |
| *YME1L1* | Forward: CCCATGTCTCTGCACAATCC | 153 bp |
|  | Reverse: ACCCCTTCACGAATGATGG |  |
| *CPT1B* | Forward: AGGATCTGGGCTTACCTAGAG | 144 bp |
|  | Reverse: ACTTGCCCACCATGACTTGA |  |
| *HADHA* | Forward: ACCGAGGACAGCAACAAGTG | 109 bp |
|  | Reverse: TCAAGCTGCCCAGTCAAGTT |  |
| *ACADM* | Forward: GGCCGTGACCCGTGTATTAT | 77 bp |
|  | Reverse: CTGCAGCATCGCCCGAA |  |
| *ATP5F1A* | Forward: TCAGTCTACGCCGCACTTAC | 174 bp |
|  | Reverse: ATGTACGCGGGCAATACCAT |  |
| *NDUFV1* | Forward: ATGAAGGTGACAGCGTGAGG | 100 bp |
|  | Reverse: TTCTTGGGTGCTGTCCGC |  |
| *CYCS* | Forward: GAGTAATAATTGGCCACTGCCT | 144 bp |
|  | Reverse: AATCAGGACTGCCCAACAAAA |  |
| *SREBP1a* | Forward: TCAGCGAGGCGGCTTTGGAGCAG | 80 bp |
|  | Reverse: CATGTCTTCGATGTCGGTCAG |  |
| *SREBP1c* | Forward: GGAGGGGTAGGGCCAACGGCCT | 80 bp |
|  | Reverse: CATGTCTTCGAAAGTGCAATCC |  |
| *METTL3* | Forward: TTGTCTCCAACCTTCCGTAGT | 145 bp |
|  | Reverse: CCAGATCAGAGAGGTGGTGTAG |  |
| *METTL14* | Forward: GCACAGACGGGGACTTCATT | 177 bp |
|  | Reverse: GCCAGCCTGGTCGAATTGTA |  |
| *FOSB* | Forward: TACTCCACACCAGGCATGAG | 265 bp |
|  | Reverse: CTTCGTAGGGGATCTTGCAG |  |
| *FOXC2* | Forward: CATGTTCGAGAACGGCAGCTT | 186 bp |
|  | Reverse: TCGCTCTTGATCACCACCTTCTT |  |
| *MEF2A* | Forward: AGCTGCTCCGGAGATACGAT | 170 bp |
|  | Reverse: TCCGCCCCATTTTCAGTCAA |  |
| *MEF2C* | Forward: AAAGGAGAGGAGGCAAACGC | 141 bp |
|  | Reverse: GGAAGTTGTACCCGTCAGCA |  |
| *MEF2D* | Forward: TGAGTTCTTAGGGTGCCTGG | 153 bp |
|  | Reverse: TGAACGGTCTGGGAACAGTG |  |
| *KLF16* | Forward: CTTTGGATGGCACTGGTGTG | 177 bp |
|  | Reverse: AGGGTTTGAGGCTAGGGACA |  |
| *RBPJ* | Forward: TACCCGCTGCGACTCTCTA | 190 bp |
|  | Reverse: AAGGCAGAAATCTGGTGCCC |  |
| *STAT5B* | Forward: ACATTAAGGCCACCCAGCTC | 105 bp |
|  | Reverse: GCCCCAGCTTGATCTTCAGT |  |
| *JDP2* | Forward: GGGGGACTGCATGGAACC | 126 bp |
|  | Reverse: CCAGGCATCATAGCAGGAGG |  |
| *EGR1* | Forward: AGTGAGCATGACCAACCCAC | 136 bp |
|  | Reverse: TGCCGCTGAGTAAATGGGAC |  |
| *MT-CO2* | Forward: CAAACCTACGCCAAAATCCA | 164 bp |
|  | Reverse: GAAATGAATGAGCCTACAGA |  |
| *KIM1* | Forward: CAGGGAAGCCGCAGAAAA | 103 bp |
|  | Reverse: GAGACACGGAAGGCAACCAC |  |
| *NGAL* | Forward: ACAAAGACCCGCAAAAGATG | 128 bp |
|  | Reverse: GCAACCTGGAACAAAAGTCC |  |
| *GAPDH* | Forward: TGACAACAGCCTCAAGAT | 103 bp |
|  | Reverse: GAGTCCTTCC ACGATACC |  |
| *β-actin* | Forward: GTGAAGGTGACAGCAGTCGGTT | 157 bp |
|  | Reverse: GAAGTGGGGTGGCTTTTAGGA |  |

**Table S5.** The primer sets for mouse qPCR

| Gene (mouse) | **Primer Sequence (5'-3')** | Product length |
| --- | --- | --- |
| *YME1L1* | Forward: AAGGCGAACTCGTCTGATCC | 96 bp |
|  | Reverse: GTAGTTGTCCGAAAGCGCAC |  |
| *KIM1* | Forward: ACATATCGTGGAATCACAACGAC | 114 bp |
|  | Reverse: ACTGCTCTTCTGATAGGTGACA |  |
| *NGAL* | Forward: GCAGGTGGTACGTTGTGGG | 95 bp |
|  | Reverse: CTCTTGTAGCTCATAGATGGTGC |  |
| *SREBP1a* | Forward: TAGTCCGAAGCCGGGTGGGCGCCGGCGCCAT | 106 bp |
|  | Reverse: GATGTCGTTCAAAACCGCTGTGTGTCCAGTTC |  |
| *SREBP1c* | Forward: ATCGGCGCGGAAGCTGTCGGGGTAGCGTC | 116 bp |
|  | Reverse: ACTGTCTTGGTTGTTGATGAGCTGGAGCAT |  |
| *METTL3* | Forward: GATAGTCCCGTGCCTACTGC | 177 bp |
|  | Reverse: TGGCGTAGAGATGGCAAGAC |  |
| *METTL14* | Forward: CTCCAGGTCGGAGTGTGAAC | 102 bp |
|  | Reverse: AACCGTTTAAACCAGCCCCT |  |
| *FOSB* | Forward: CAGCTAAGTGCAGGAACCGT | 218 bp |
|  | Reverse: ACTTGAACTTCACTCGGCCA |  |
| *FOXC2* | Forward: CGGCGCTTCAAGAAGAAGGA | 154 bp |
|  | Reverse: CCTCGCTCTTAACCACGACTT |  |
| *MEF2A* | Forward: GCGGAGACTCGGAATTGCAT | 114 bp |
|  | Reverse: CAGGGCTGCCGTTGAAATTG |  |
| *MEF2C* | Forward: GAGCCGGACAAACTCAGACA | 101 bp |
|  | Reverse: GGCTGTGACCTACTGAATCGT |  |
| *MEF2D* | Forward: AGATCTGAACAATGCCCAGC | 194 bp |
|  | Reverse: CTGCAGGTGAACTGAAGGCT |  |
| *KLF16* | Forward: GTCCTCTCTGACTGCTGGTG | 132 bp |
|  | Reverse: AACTCAGGGACCCTAACCCA |  |
| *RBPJ* | Forward: CACCTGCGTGTAAGGGTCAT | 185 bp |
|  | Reverse: GGCACATCCCACAGAAGACA |  |
| *STAT5B* | Forward: AGAGAGCGTGCGGGACC | 188 bp |
|  | Reverse: AGGCTTGGCTTTCGATCCAC |  |
| *JDP2* | Forward: TACGCTGACATCCGCAACAT | 100 bp |
|  | Reverse: CGTCTAGCTCACTCTTCACGG |  |
| *EGR1* | Forward: AGTGATGAACGCAAGAGGCA | 121 bp |
|  | Reverse: TAGCCACTGGGGATGGGTAA |  |
| *ACADM* | Forward: TCAAGATCGCAATGGGTGCT | 139 bp |
|  | Reverse: TGCTCCACTAGCAGCTTTCC |  |
| *NDUFV1* | Forward: AAGCCATCGCTCGTCTCATT | 115 bp |
|  | Reverse: TCTCCCTTCACAAATCGGGC |  |
| *FASN* | Forward: CTGCCTTCGGTTCAGTCTCTT | 123 bp |
|  | Reverse: CAGAATGGCACACCCTCCAA |  |
| *ACC1* | Forward: GGCGGATATCTGCTGAGACAA | 114 bp |
|  | Reverse: GGGAGTGCTGGTTTAGCTCC |  |
| *β-actin* | Forward: TGTTACCAACTGGGACGACA | 165 bp |
|  | Reverse: GGGGTGTTGAAGGTCTCAAA |  |

**Table S6.** The sequence sets for siRNA

| siRNA (human) | Primers |
| --- | --- |
| *si* *YME1L1-1* | Forward: 5′- GUAUCGACAUCAUUCAAGAdTdT |
|  | Reverse: 5′- UCUUGAAUGAUGUCGAUACdTdT |
| *si* *YME1L1-2* | Forward: 5′- GGACCUUGGAUUAUCUGAAdTdT |
|  | Reverse: 5′- UUCAGAUAAUCCAAGGUCCdTdT |
| *siMETTL3-1* | Forward: 5′- CAGUUCGUUAGUCUCUGGUdTdT |
|  | Reverse: 5′- ACCAGAGACUAACGAACUGdTdT |
| *siMETTL3-2* | Forward: 5′- CUCCAACCUUCCGUAGUGAdTdT |
|  | Reverse: 5′- UCACUACGGAAGGUUGGAGdTdT |
| *siIGF2BP1-1* | Forward: 5′-GGGAAGAAUCUAUGGCAAAdTdT |
|  | Reverse: 5′-UUUGCCAUAGAUUCUUCCCdTdT |
| *siIGF2BP1-2* | Forward: 5′-GGGCCGAGCAGGAAAUAAUdTdT |
|  | Reverse: 5′-AUUAUUUCCUGCUCGGCCCdTdT |
| *siIGF2BP2-1* | Forward: 5′- CAAACUAGCCGAAGAGAUUdTdT |
|  | Reverse: 5′- AAUCUCUUCGGCUAGUUUGdTdT |
| *siIGF2BP2-2* | Forward: 5′-GAUAUGCUGGCUGUUAACAdTdT |
|  | Reverse: 5′-UGUUAACAGCCAGCAUAUCdTdT |
| *siIGF2BP3-1* | Forward: 5′- GGGAAGAAUUUAUGGAAAAdTdT |
|  | Reverse: 5′- UUUUCCAUAAAUUCUUCCCdTdT |
| *siIGF2BP3-2* | Forward: 5′-GAAUCUUCAAGCACAUUUAdTdT |
|  | Reverse: 5′-UAAAUGUGCUUGAAGAUUCdTdT |
| *siOPA1-1* | Forward: 5′-GUGGCCCUAUUUAAAGAUAdTdT |
|  | Reverse: 5′-UAUCUUUAAAUAGGGCCACdTdT |
| *siOPA1-2* | Forward: 5′- CCGGACCUUAGUGAAUAUAdTdT |
|  | Reverse: 5′- UAUAUUCACUAAGGUCCGGdTdT |
| *NC* | Forward: 5′-UUCUCCGAACGUGUCACGUdTdT |
|  | Reverse: 5′-ACGUGACACGUUCGGAGAAdTdT |

**Table S7.** The sequence sets for sgRNA

| sgRNA (human) | Primers |
| --- | --- |
| *sgEGR1* | 5′-GCAAGCGTAAGGGCGTTCGT-3′ |
| *sgSREBP1c* | 5′-GCCATGGATTGCACTTTCGA-3′ |
| *sgFOSB* | 5′-GTCGTAGGGGTCGACGACCG-3′ |

**Table S8.** The primer sets for PCR amplification of the human *YME1L1* promoter region

| Fragment | Primers (The restriction enzyme KpnⅠ and XhoⅠ cutting sites are underlined) |
| --- | --- |
| -2000 to +200 | Reverse: 5′-AGAACATTTCTCTATCGATAGGTACCCTGTGGTAGAA AAATTTCGACGATTCTGCACGT-3′ |
|  | Reverse: 5′-AGCTTACTTAGATCGCAGATCTCGAGCCCTCAGCGAC CTCACCCGCCTGCCGAAACTGT-3′ |
| -1000 to +200 | Forward: 5′-AGAACATTTCTCTATCGATAGGTACCACAGCATACAG CGGCCCCGC-3′ |
|  | Reverse: 5′- AGCTTACTTAGATCGCAGATCTCGAGCCCTCAGCGAC CTCACCCGC-3′ |
| -700 to +200 | Forward: 5′-AGAACATTTCTCTATCGATAGGTACCGAATCGCAGGAT TTGAAGAG-3′ |
|  | Reverse: 5′-AGCTTACTTAGATCGCAGATCTCGAGCCCTCAGCGACC TCACCCGC-3′ |
| -400 to +200 | Forward: 5′-AGAACATTTCTCTATCGATAGGTACCCACTAAATTGTA GCAAAAGAC-3′ |
|  | Reverse: 5′-AGCTTACTTAGATCGCAGATCTCGAGCCCTCAGCGACC TCACCCGC-3′ |
| -150 to +200 | Forward: 5′-AGAACATTTCTCTATCGATAGGTACCAAGAAAAAGTCC AAAGAGGG-3′ |
|  | Reverse: 5′-AGCTTACTTAGATCGCAGATCTCGAGCCCTCAGCGACC TCACCCGC-3′ |

**Table S9.** The primer sets for ChIP

| Fragment | Primer Sequence (5'-3') | Product length |
| --- | --- | --- |
| YME1L1 (-302~-171) | Forward: GTGGCGGGCACCTGTAA | 132 bp |
|  | Reverse: CGGAGTTTCGTTCTTTCG |  |
| YME1L1 (-1358~-1161) | Forward: GCTGATGTCGCCCCAAAAAG | 198 bp |
|  | Reverse: GCAGAAAGGCGGCAAATTGT |  |
